# Designing Water-Soluble Macromolecules for Biomedical Use: PEG Chains versus Amino Acids—A Case Study in MRI Contrast Agents

**DOI:** 10.1021/acspolymersau.5c00162

**Published:** 2026-02-17

**Authors:** Yufei Wu, Carlos Caro, Esther Matamoros, Jesús David Urbano-Gámez, Silvia Lope-Piedrafita, Yolanda Vida, María Luisa García-Martín, José Vidal-Gancedo, Vega Lloveras

**Affiliations:** † 54449 Institut de Ciència de Materials de Barcelona, ICMAB-CSIC, Campus UAB, 08193 Bellaterra, Spain; ‡ Biomedical Magnetic Resonance Laboratory-BMRL, Andalusian Public Foundation Progress and Health-FPS, Avda. Américo Vespucio, 15, 41092 Seville, Spain; § 582139 Instituto de Investigación Biomédica de Málaga y Plataforma en Nanomedicina−IBIMA, Plataforma Bionand, ParqueTecnológico de Andalucía, 29590 Málaga, Spain; ∥ Departamento de Química Orgánica, 16752 Universidad de Málaga, 29071 Málaga, Spain; ⊥ Departament de Bioquímica i Biologia Molecular, Unitat de Biofísica, Facultat de Medicina, Universitat Autònoma de Barcelona, 08193 Bellaterra, Spain; # Networking Research Center on Bioengineering, Biomaterials and Nanomedicine, CIBER-BBN, Instituto de Salud Carlos III, Campus UAB, 08913 Bellaterra, Spain; ∇ Networking Research Center on Bioengineering, Biomaterials and Nanomedicine, CIBER-BBN, ParqueTecnológico de Andalucía, 29590 Málaga, Spain

**Keywords:** biomaterials, radical dendrimers, water-soluble, molecular dynamics, MRI contrast agents, relaxivity, dynamic contrast-enhanced MRI

## Abstract

Understanding how macromolecular architecture and interfacial hydration govern magnetic relaxation is crucial for the rational design of next-generation Magnetic Resonance Imaging (MRI) contrast agents. Here, we compare two water-solubilization strategies for organic radical dendrimers: using short-chain poly­(ethylene glycol) (PEG) linkers versus amino acid-derived carboxylate sodium salt linkers. By synthesizing third- and fourth-generation dendrimers functionalized with these respective groups (denoted as GnPEG and GnNa, where *n* = 3, 4), we achieved well-controlled branching and radical loading. This design enables a systematic investigation into how linker chemistry governs molecular flexibility, interfacial hydration, and spin exchange efficiency. *In vitro* MRI revealed that the GnNa series exhibits higher longitudinal relaxivity (*r*_1_) than the GnPEG counterparts. Electron paramagnetic resonance (EPR) spectroscopy and molecular dynamics (MD) simulations were employed to elucidate the factors underlying these differences. The combined experimental and computational analysis indicates that amino acid carboxylate linkers enhance hydration and hydrogen bonding at the dendrimer–solvent interface relative to PEG-linked analogues, correlating with improved relaxivity. *In vitro* and *in vivo* MRI studies demonstrated that G4Na achieves an *r*_1_ of 5.01 mM^–1^·s^–1^ per molecular entity at 7 T—comparable to or exceeding commercial Gd^3+^-based agents—while maintaining physiological stability, rapid renal clearance, and effective tumor imaging capability. These findings demonstrate that fine-tuning dendrimer–solvent interface interactions through amino acid carboxylate linkers provides a robust strategy for optimizing water accessibility and relaxation efficiency in macromolecular MRI agents. This work establishes a clear structure–interface–function relationship that can guide the future design of biomacromolecules or biocolloidal architectures for biomedical imaging.

## Graphical abstract

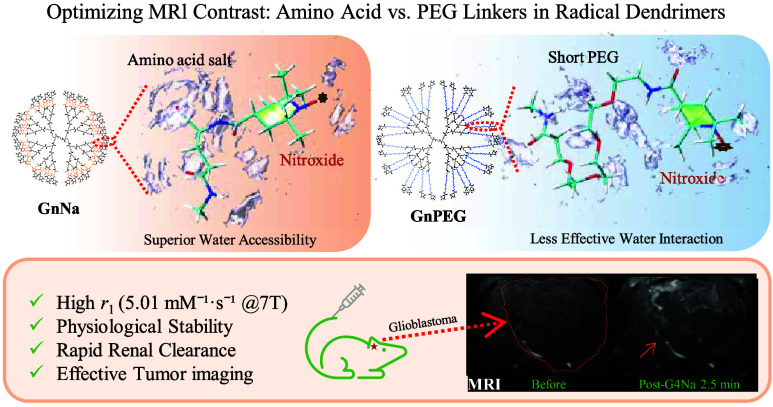

## 1 Introduction

Magnetic Resonance Imaging (MRI) is a medical imaging technique that uses magnetic fields and radio waves to create detailed images of internal organs and tissues without exposing patients to ionizing radiation, distinguishing it from other imaging modalities.^1^ However, conventional MRI scans often suffer from limited intrinsic contrast, necessitating the use of contrast agents to enhance the visibility of specific anatomical structures and pathological changes.^2^

The most widely used agents are Gadolinium-based paramagnetic contrast agents (GBCAs).^3^ These agents, containing the metal ion Gadolinium (Gd^3+^), exert a strong influence on the relaxation times of water molecules in the body, both longitudinal (*T*_1_) and transverse (*T*_2_).^4^ GBCAs shorten both *T*_1_ and *T*_2_ relaxation times; however, their predominant effect is on *T*_1_, leading to increased signal intensity in *T*_1_-weighted images. This makes them primarily classified as positive contrast agents. Conversely, negative contrast agents, such as superparamagnetic iron oxide nanoparticles (SPIONs),^5–8^ predominantly reduce signal intensity by shortening *T*_2_ relaxation time, resulting in darker contrast-enhanced tissues on *T*_2_-weighted images.

While GBCAs are generally well tolerated, patients with severe renal impairment or those undergoing dialysis are at risk of developing a life-threatening condition known as nephrogenic systemic fibrosis (NSF).^9^ Additionally, growing evidence suggests that gadolinium ions can accumulate in the brain and other organs in patients with normal renal function, raising concerns about long-term toxicity.^10,11^ These safety issues highlight the urgent need to develop safer alternatives,^12,13^ with recent strategies focusing on macrocyclic confinement,^14^ peptide targeting,^15^ and relaxation enhancement via molecular interactions^4^ to improve safety.

Iron oxide contrast agents like Ferumoxytol offer an alternative for MRI, but serious allergic risks and subsequent FDA warnings have greatly limited their clinical application.^16,17^ Another research avenue focuses on the development of metal-free contrast agents. Chemical Exchange Saturation Transfer (CEST) agents facilitate disease diagnosis and metabolic assessment by detecting specific molecules via proton exchange; however, their detection limits typically fall in the millimolar range—orders of magnitude higher than GBCAs.^18^ Similarly, while ^19^F MRI can identify subtle biological changes for early detection and personalized therapy, it lacks the signal amplification inherent to relaxation probes, necessitating high local concentrations of fluorine nuclei.^19^ Furthermore, hyperpolarized ^13^C imaging is constrained by rapid signal decay, extremely short imaging windows, and the need for costly, specialized instrumentation.^20^ These alternatives rely on unconventional imaging mechanisms and frequently face challenges regarding insufficient sensitivity or poor water solubility.^12^ In contrast, organic radical agents, such as nitroxides, generate MRI contrast via standard water relaxation mechanisms. As they require no specialized equipment or techniques, they offer a more straightforward pathway for rapid clinical translation.^21^

In the early 1980s, nitroxyl radicals were investigated as *T*_1_ contrast agents for MRI.^22,23^ Nitroxides have the advantages of high *in vitro* stability, low toxicity, and structural tunability.^24^ However, nitroxides have only one unpaired electron, which results in low relaxivity^25^ and rapid reduction *in vivo*.^26^ One strategy that has been shown to achieve higher molecular relaxivity and radical protection is to conjugate them with macromolecules, such as linear^27^ or cross-linked polymers,^28,29^ star polymers,^30^ self-assembled nanoparticles,^31,32^ micelles,^33^ viruses^34^ and proteins,^35^ etc.

Water solubility, stability, and biocompatibility are critical for materials used as MRI contrast agents *in vivo*.^36^ In addition, it is also essential a strict control over their molecular structure that guarantees reproducibility, along with the benefits of rapid synthesis and low production cost. In this context, dendrimers—highly branched, monodisperse macromolecules with well-defined architectures—emerge as ideal candidates, offering significant advantages for studying relaxation processes.^37,38^ When fully functionalized with organic radicals, we term them radical dendrimers.^39–49,37,50^ Common scaffolds include polyamidoamine (PAMAM), polypropylenimine (PPI), and polyester (bis-MPA) dendrimers.^51–53^ However, PAMAM and PPI are prone to structural defects caused by retro-Michael reactions and intramolecular cyclization.^54^ Despite a relatively monodisperse distribution (PDI ∼ 1.01), PAMAM often lacks terminal arms or forms intramolecular rings, compromising its structural integrity.^55^ Even highly purified G5 PAMAM contains a significant proportion of defective structures, with perfect structures constituting less than 0.01% in some cases, depending on synthesis and purification methods. Similarly, bis-MPA dendrimers are susceptible to hydrolysis under physiological conditions, which limits their suitability for *in vivo* applications.^56^

Amino-terminated polyamide dendrimers present a promising alternative, offering efficient and low-cost synthesis, simple purification, and minimal steric hindrance.^57^ For example, fifth-generation dendrimers (G5–64NH_2_), containing 64 amino termini, can be synthesized in high amounts with good yields. In this study, third-generation (G3–16NH_2_) and fourth-generation (G4–32NH_2_) polyamide dendrimers were selected as scaffolds for the synthesis of radical dendrimers (Figure 1), using the stable nitroxyl radical 2,2,6,6-tetramethylpiperidine 1-oxyl (TEMPO).

**Figure 1:**
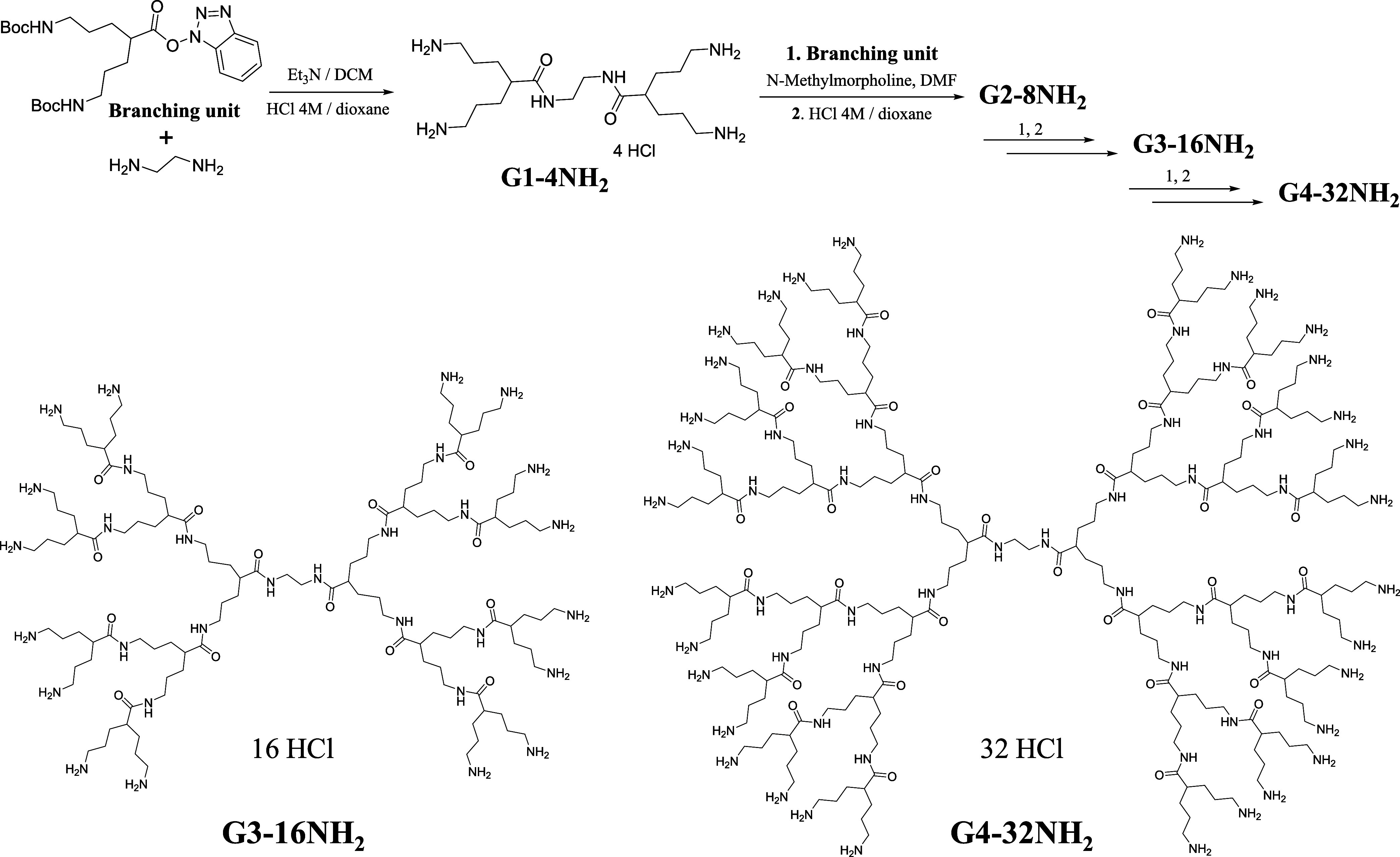
Synthesis and structures of G3–16NH_2_ and G4–32NH_2_ dendrimers.

A common strategy to achieve water solubility in polymers or dendrimers involves the statistical incorporation of hydrophilic PEG chains. However, when only a portion of the branches are functionalized with radicals and the remainder with PEG, it becomes challenging to precisely control radical loading—complicating structural definition as well as relaxivity calculations.

In this work, we use PEG chains as linkers between the dendrimer branches and the radicals, enabling full radical incorporation while maintaining high water solubility. In addition, this approach is compared with the use of another hydrophilic linker, amino acids. In this case, water solubility is provided by the carboxylic acid groups, which are converted to their sodium salt forms.^44^ Both linker systems—PEG chains and amino acids—allow complete functionalization of all dendrimer branches with radicals and significantly enhance aqueous solubility. This dual-linker strategy, enables a direct comparison of the two linker systems using the same dendrimer platform, providing insight into their effects on synthetic efficiency, water solubility, and MRI-relevant properties such as relaxivity, with particular emphasis on the dendrimer–water interface.

By combining experimental data with molecular dynamics (MD) simulations, the study examined the relationship between structural and dynamics parameters and the relaxivity behavior of the two types of water-soluble radical dendrimers at the dendrimer–water interface. Finally, the *in vivo* pharmacokinetics and tumor imaging capabilities of the most promising candidate are assessed in mice to evaluate its potential as an MRI contrast agent.

## 2 Materials and Methods

### 2.1 Chemicals

All reactions were performed using commercially available reagents without further purification unless stated otherwise. Sodium hydrogen carbonate (NaHCO_3_), sodium chloride (NaCl), magnesium sulfate (MgSO_4_), hexafluorophosphate azabenzotriazole tetramethyl uronium (HATU), 4-carboxy-2,2,6,6-tetramethylpiperidine 1-oxyl (TEMPO–COOH), trifluoroacetic acid (TFA), and *N*,*N*-diisopropylethylamine (DIEA) were obtained from Sigma-Aldrich. Additional reagents included 15-(Boc-amino)-4,7,10,13-tetraoxapentadecanoic acid (BocNH-(PEG)_4_-COOH) and (*S*)-4-((*tert*-butoxycarbonyl)­amino)-5-methoxy-5-oxopentanoic acid (Boc-Glu-OMe-COOH) were obtained from CymitQuímica. Dichloromethane (DCM) was purified by distillation over calcium hydride (CaH_2_). Other solvents, such as *n*-hexane, dimethyl sulfoxide (DMSO), diethyl ether (Et_2_O), and methanol (MeOH), were used as received. Water was purified using a Milli-Q water purification system (Millipore). Dialysis was performed using dialysis bags with a molecular weight cutoff (MWCO) of 100–500 Da (Thermo Fisher Scientific Inc.). Lyophilization was conducted using a Telstar-LYO QUEST lyophilizer. Dulbecco’s Modified Eagle Medium (DMEM), Fetal Bovine Serum (FBS), l-glutamine, and penicillin/streptomycin solution were obtained from Gibco.

### 2.2 Animals

Balb/c mice (for determination of pharmacokinetics in healthy animals) and SCID mice (for tumor targeting experiments in glioma-bearing mice) weighing ∼20 g were obtained from Charles River. *In vivo* experiments were conducted following Spanish and European Guidelines for Care and Use of Laboratory Animals (R.D. 53/2013 and 2010/62/UE) and approved by our local ethical committee (IBIMA Plataforma BIONAND) and the Highest Institutional Ethical Committee (Andalusian Government, accreditation number 14/09/2021/129). Further information about animal housing and care is provided in the SI.5.

### 2.3 Methods

#### 2.3.1 Proton Nuclear Magnetic Resonance (^1^H NMR)

^1^H NMR analyses were conducted at 25 °C using a Bruker Advance III-400 MHz spectrometer. Deuterated solvents, including deuterated water (D_2_O), deuterated methanol (CD_3_OD), and deuterated dimethyl sulfoxide (DMSO-*d*_6_) were used.

#### 2.3.2 Fourier Transform Infrared (FT-IR)

FT-IR spectra were acquired using a JASCO 4700 LE spectrometer equipped with an Attenuated Total Reflectance (ATR) accessory. The analysis was performed over the spectral range of 400–4000 cm^–1^ with a resolution of 4 cm^–1^.

#### 2.3.3 Size Exclusion-High-Performance Liquid Chromatography (SEC-HPLC)

SEC-HPLC was performed on an Agilent 1260 Infinity II system with a diode array detector. Separation was achieved using a PSS Suprema precolumn (10 μm, 8 mm × 50 mm) and a PSS Suprema analytical column (10 μm, 100 Å, 8 mm × 300 mm). The mobile phase consisted of 0.25 mM LiCl aqueous solution, and samples (1 mg/mL) were prepared in the eluent and filtered through a 0.22 μm PTFE filter. The flow rate was maintained at 0.5 mL/min.

#### 2.3.4 Electron Paramagnetic Resonance (EPR)

EPR spectra were recorded on a Bruker ELEXSYS E-500 spectrometer (X-band, 9.4 GHz) with a ST8911 microwave cavity, variable temperature unit, and field frequency lock system. Modulation amplitude and microwave power were optimized to avoid saturation and spectral broadening. Liquid samples were degassed with argon, and measurements were performed using 4 mm quartz tubes for organic solutions or flat quartz cells for aqueous solutions. The EPR spectra were simulated by fitting the experimental data using MATLAB R2024a in conjunction with the EasySpin toolbox (version 6.0.2).^58^ Spectral and dynamic parameters were obtained by fitting the simulated EPR spectra to the experimental data using a least-squares fitting algorithm. The EasySpin function “chili” was employed for the spectral simulations. The EPR spectra were decomposed into two components: one representing isolated radicals (“free” component or noninteracting component) and another representing exchange-coupled radicals (exchange component). The “free” component was simulated by varying the line width and rotational correlation time. The exchange component accounted for spin exchange between closely adjacent radicals in solution, with both line width and rotational correlation time as adjustable parameters as well. The fitting methodology followed procedures described in our previous studies.^49,50^ A least-squares fitting algorithm (Nelder–Mead simplex method) was employed to globally minimize the root-mean-square deviation between experimental and simulated spectra. Convergence was confirmed when the iterative change in error fell below a preset threshold.

#### 2.3.5 Molecular Dynamics (MD)

MD simulations were performed in explicit water using GROMACS 2019.2 program.^59^ Detailed parameters and protocols are provided in the Supporting Information (SI.4).

#### 2.3.6 Cytotoxicity

##### 2.3.6.1 Cell Culture

Vero cells were routinely cultured in DMEM + 10% FBS growth medium at 37 °C and 5% CO_2_ (standard culture conditions). Vero cells were on passage number 17 for the experiment.

##### 2.3.6.2 Material Handling

The dendrimers were supplied in powder form, dispersed in DMEM supplemented with 1% FBS, and subsequently filtered through 0.22 μm PES syringe filters to ensure sterility. A final concentration of 2 mM per radical unit was achieved in all cases. The samples were then serially diluted by half, five times, resulting in a final concentration of 0.06 mM. *Resazurin cell viability assay*: Vero cells were seeded in 96 well plates at 15 × 10^3^ cells/well and incubated for 24 h at 37 °C and 5% CO_2_. After the initial incubation, the radical dendrimers dispersed in DMEM + 1% FBS and controls were added to the cells (100 μL) in the previously stated concentrations. 24 h after exposure to the samples, media was removed from all samples and resazurin 45 μM in DMEM was added to all samples (100 μL). Samples were incubated for 4 h at 37 °C and 5% CO_2_. Afterward, 95 μL of all wells were transferred to a black 96-well plate for fluorescence measurements (λ_Ex_ = 545 nm; λ_Em_ = 590 nm).

#### 2.3.7 Cell Culture and Tumor Implantation

C6 cells were grown in DMEM (supplemented with l-glutamine (2 mM)), FBS (10%), and penicillin/streptomycin (1%). Cells were cultured under standard conditions at 37 °C in a 5% CO_2_ incubator. A total of 10^5^ C6 cells were suspended in 4 μL of culture medium and inoculated into the right caudate nucleus (2.3 mm to the right of the bregma and 3.3 mm from the cranial surface) of SCID mice, as described previously by some of us.^60^

#### 2.3.8 Magnetic Resonance Imaging (MRI)

*In vitro* studies: MRI experiments were conducted on Bruker BioSpec 70/30 system, featuring a 7.0 T horizontal-bore superconducting magnet equipped with actively shielded gradients (B-GA12 gradient coil inserted into a B-GA20S gradient system) and a quadrature 72 mm inner diameter volume coil. The relaxivity values (*r*_1_ and *r*_2_) were determined by linear regression of the relaxation rates (1/*T*_1_ and 1/*T*_2_) versus concentration. This SD represents the standard error of the slope derived from the linear regression fit, providing a statistical measure of the reliability of the relaxivity determination. *In vivo* studies: Dendrimers were administered intravenously at 0.072 mmol/kg per dendrimer and followed by MRI using a 9.4 T Bruker BioSpec system equipped with 400 mT/m gradients and a 40 mm quadrature birdcage resonator or a surface coil (designed for mouse brain imaging). MRI data acquisition was managed using ParaVision 7.0 software (Bruker BioSpin, Ettlingen, Germany) on a Linux platform. Throughout the procedure, respiration and body temperature were continuously monitored using a Small Animal Monitoring and Gating System (PC-SAM 32 v8.02 software). For details on experimental design, sequences used, and data computation, see SI.8.

#### 2.3.9 Histology

After the sacrifice of the animals, tissues were collected and stained. Histological analysis was conducted using a LEICA APERIO VERSA 200 in light microscopy mode. Tissue architecture was evaluated with Hematoxylin and Eosin (H&E) staining. As controls, mice were injected with 0.9% NaCl saline. Detailed protocols for this procedure are provided in the SI.7.

## 3 Results and Discussion

### 3.1 Synthesis of Radical Dendrimers GnNa and GnPEG (*n* = 3, 4)

The synthesis commenced with amino-terminated polyamide dendrimers, G3–16NH_2_ and G4–32NH_2_ (denoted as Gn-NH_2_, *n* = 3, 4), prepared according to literature methods (Figure 1).^57^ For hydrophilic linkers, two strategies were explored. As the amino acid–based linker, we employed a derivative of glutamic acid (Glu). For the PEG-based approach, we aimed to use the shortest PEG chain that would still provide sufficient water solubility and facilitate handling during the coupling reaction. Although longer PEG chains such as PEG6 offer improved solubility, their use was limited by steric hindrance at the dendrimer periphery, which restricted the reaction to approximately half of the available amino groups. As a result, PEG4 was selected as the optimal compromise. Following the grafting of these linkers and subsequent conjugation of TEMPO radicals (Figure 2a), two series of water-soluble radical dendrimers were obtained: the sodium carboxylate-functionalized series (denoted as GnNa) and the PEG-linked series (denoted as GnPEG), as illustrated in Figure 2b.

**Figure 2:**
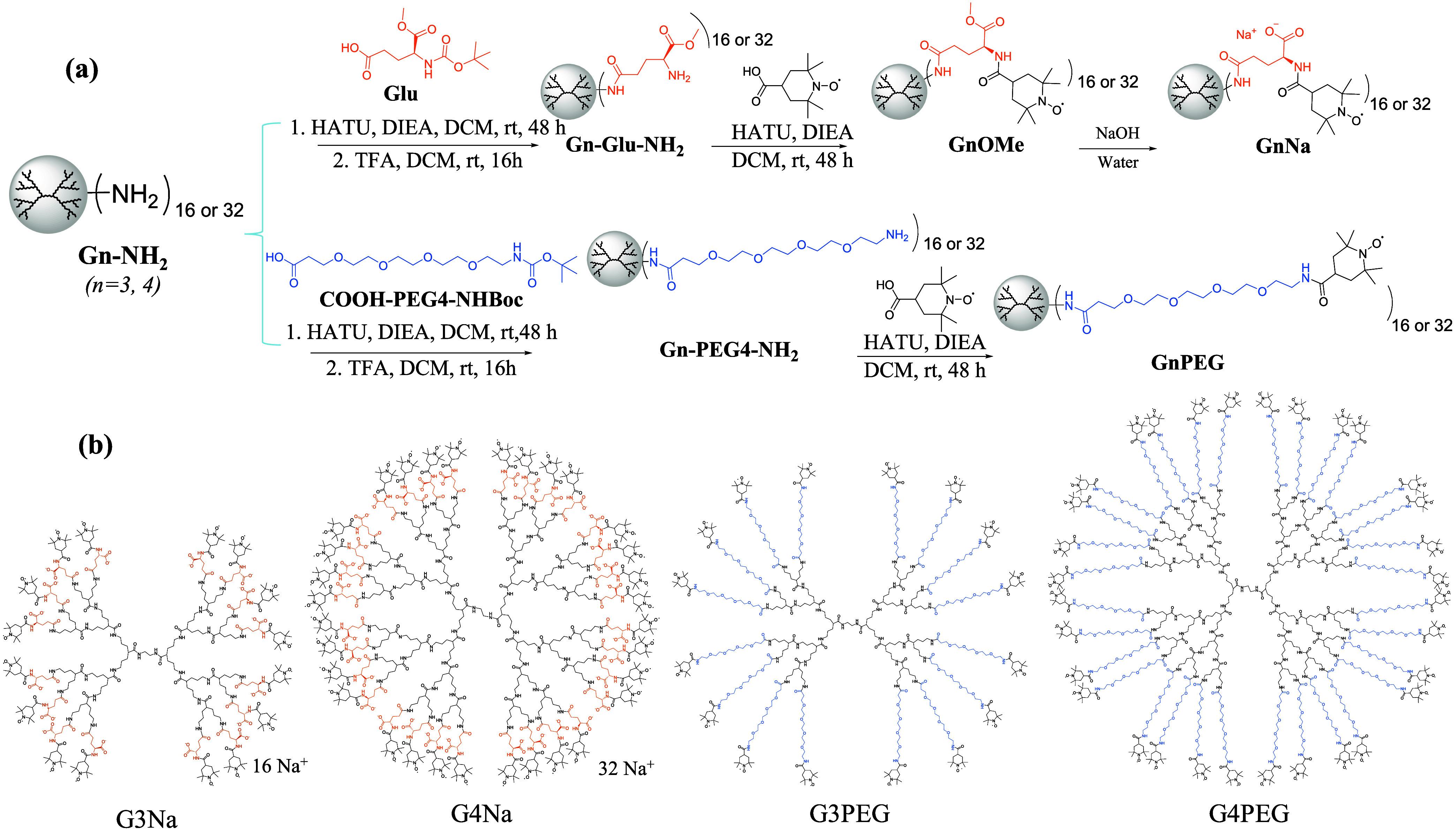
(a) Synthesis pathway to obtain GnNa and GnPEG and (b) structures of GnNa and GnPEG.

The first step of this process involved an amidation reaction, where amine-ended dendrimers (Gn-NH_2_) reacted with the carboxylic acid of the linkers (Glu and PEG4 derivatives). The carboxylic groups of the linkers were activated using HATU in the presence of excess DIEA, followed by the addition of Gn-NH_2_ for a 48 h reaction. Subsequently, the *N*-Boc-protected products (Figure 2a) were obtained through precipitation in *n*-hexane/Et_2_O after washing with water.

In the second step, the obtained products underwent *N*-Boc deprotection using TFA/DCM (1:1). The reaction mixture was dripped into *n*-hexane/Et_2_O to easily obtain products Gn-Glu-NH_2_ and Gn-PEG4-NH_2_. Column chromatography was unnecessary, and the overall yield for these two steps was approximately 80%.

Subsequently, the same amidation method as in the first step was employed to couple TEMPO–COOH to Gn-Glu-NH_2_ and Gn-PEG4-NH_2_, yielding GnOMe and GnPEG with a yield usually exceeding 90%. For GnOMe, an excess of NaOH/H_2_O solution was used to convert the methyl ester to a carboxylate sodium salt. The ester-carboxylate conversion was monitored using FT-IR spectroscopy (see Figure S1), and the product (GnNa) was then purified by dialysis with a yield of approximately 95%. For detailed synthetic procedures, refer to SI.1. Figure 2b shows the final structures of GnNa and GnPEG. From a translational perspective, the synthetic approach used here is highly scalable and reproducible. In contrast to many functionalized nanomaterials that require complex purification, the dendrimer conjugates were purified using straightforward precipitation and dialysis methods that are easily scaled and avoid the need for column chromatography.

The synthesized dendrimers exhibited excellent water solubility at room temperature, with approximate solubilities as follows: G3Na (550 mg/mL), G4Na (320 mg/mL), G3PEG (110 mg/mL), and G4PEG (60 mg/mL). Overall, GnNa radical dendrimers exhibited significantly higher solubility than their GnPEG counterparts, and third-generation dendrimers were more soluble than fourth-generation ones. Among all the compounds, G3Na showed the highest solubility.

### 3.2 Characterization

Characterization was primarily performed using FT-IR, ^1^H NMR, and electron paramagnetic resonance (EPR) spectroscopy, along with SEC-HPLC.

The structure of the dendrimers was characterized by ^1^H NMR spectroscopy. The number of hydrogen atoms was determined from the relative integrals of the ^1^H resonances, which matched the theoretical proton count (Figure 3), thereby confirming the overall structure and the successful attachment of all TEMPO radical units. Gn-Glu-NH_2_ and Gn-PEG4-NH_2_ dendrimers could be directly analyzed by ^1^H NMR (Figures S2–S5). However, due to the paramagnetic nature of the TEMPO radicals, the GnNa and GnPEG dendrimers required chemical reduction to their corresponding hydroxylamines prior to NMR analysis. This reduction was performed using an excess of reducing agent (phenylhydrazine or ascorbic acid) in a suitable deuterated solvent, employing a 20-fold molar excess relative to the radical units.

**Figure 3:**
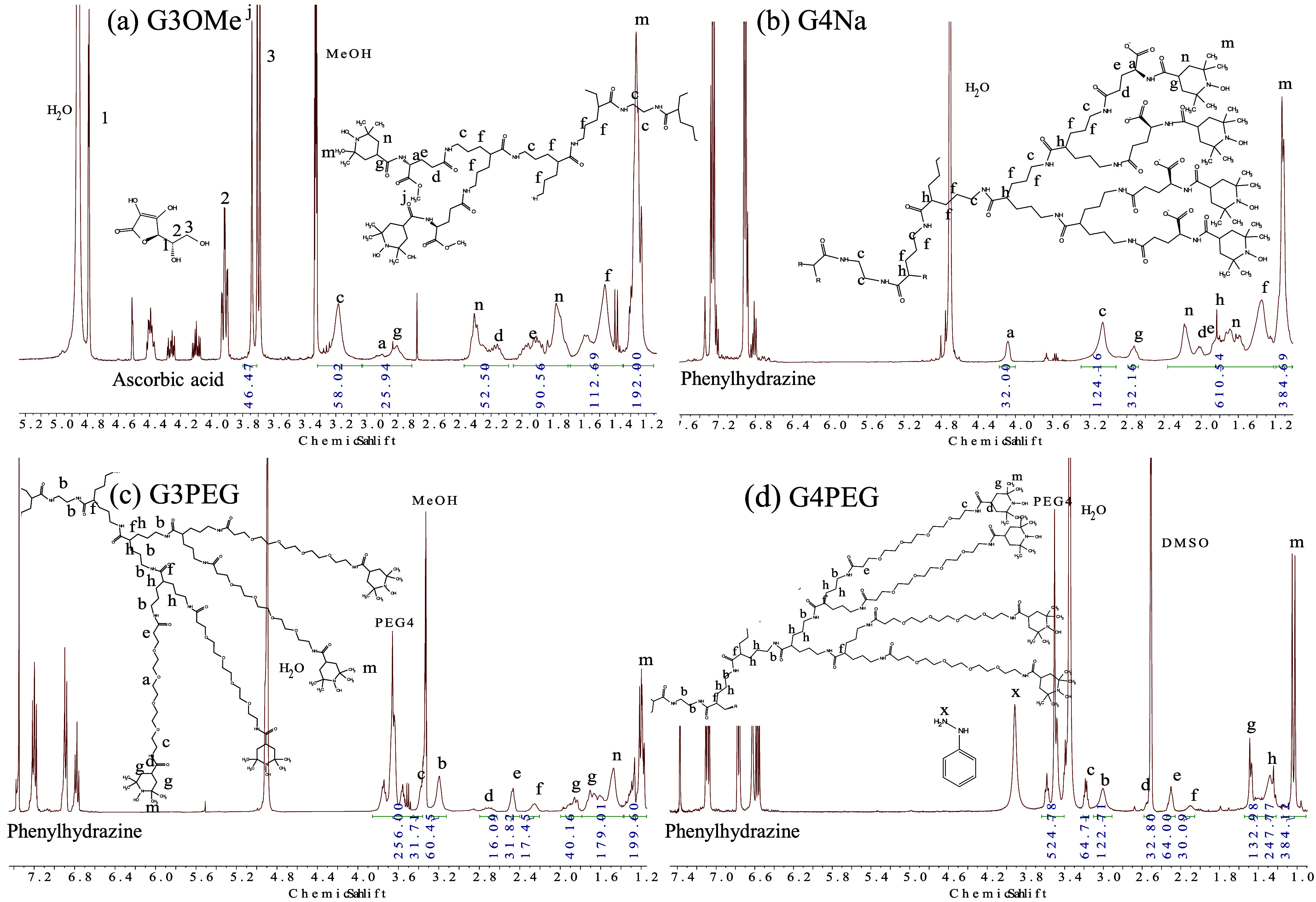
^1^H NMR spectra of the following dendrimers and the corresponding deuterated solvents used: (a) G3OMe, CD_3_OD + ascorbic acid; (b) G4Na, D_2_O + phenylhydrazine; (c) G3PEG, CD_3_OD + phenylhydrazine; (d) G4PEG, DMSO-*d*_6_ + phenylhydrazine.

The SEC-HPLC analysis revealed monodisperse peaks for all four final radical dendrimers (Figure 4), indicating high structural uniformity. Within each dendrimer type, higher-generation molecules exhibited shorter elution times than their lower-generation counterparts. This is consistent with the expected trend that higher-generation dendrimers have larger and more compact structures due to the increased branch density. Notably, GnNa displayed shorter elution times and stronger detector signals compared to GnPEG. This difference may be attributed to variations in hydrodynamic size or differential interactions with the aqueous LiCl eluent. In particular, PEG-modified dendrimers may experience stronger interactions with the eluent, potentially leading to increased retention or reduced signal intensity.^61^

**Figure 4:**
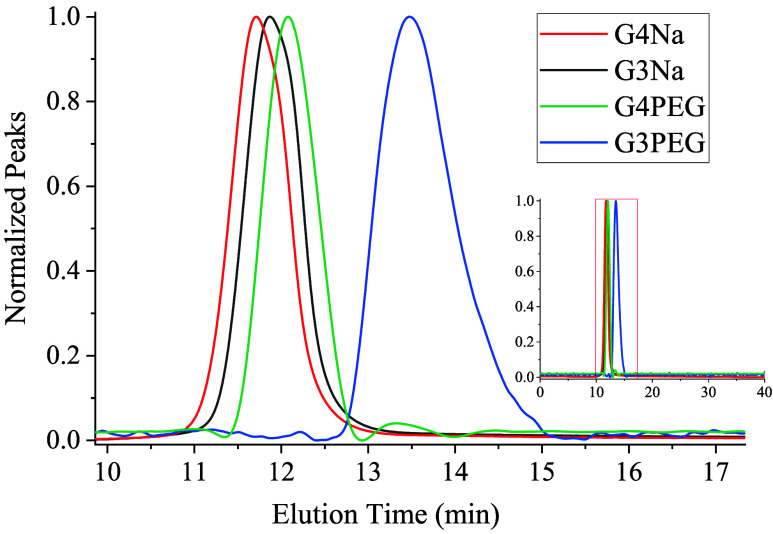
Normalized SEC-HPLC chromatogram of the final radical dendrimers.

### 3.3 EPR Analysis

EPR analysis was essential both for characterization and for extracting detailed dynamic information from spectral simulations. In particular, it enabled the determination of the rotational correlation time (τ_corr_), a key parameter that can influence relaxivity.

EPR spectra of the four radical dendrimers were first recorded in aqueous solution at 300 K, maintaining a constant concentration of 0.63 mM (per TEMPO unit) (Figure 5a). The spectra predominantly exhibit the characteristic three-line pattern of nitroxides, and all dendrimers displayed similar integrated signal areas, indicating a consistent number of radical units across generations. However, compared to free TEMPO in low-viscosity solvents, the spectra display broader lines, indicating spin exchange and dipolar interactions as well as restricted rotational mobility.^62^ This line broadening reflects the covalent attachment of nitroxide units to the dendrimer scaffold and their close spatial proximity, which results in increased spin–spin interactions, more pronounced in the fourth generation than in the third.^50^

**Figure 5:**
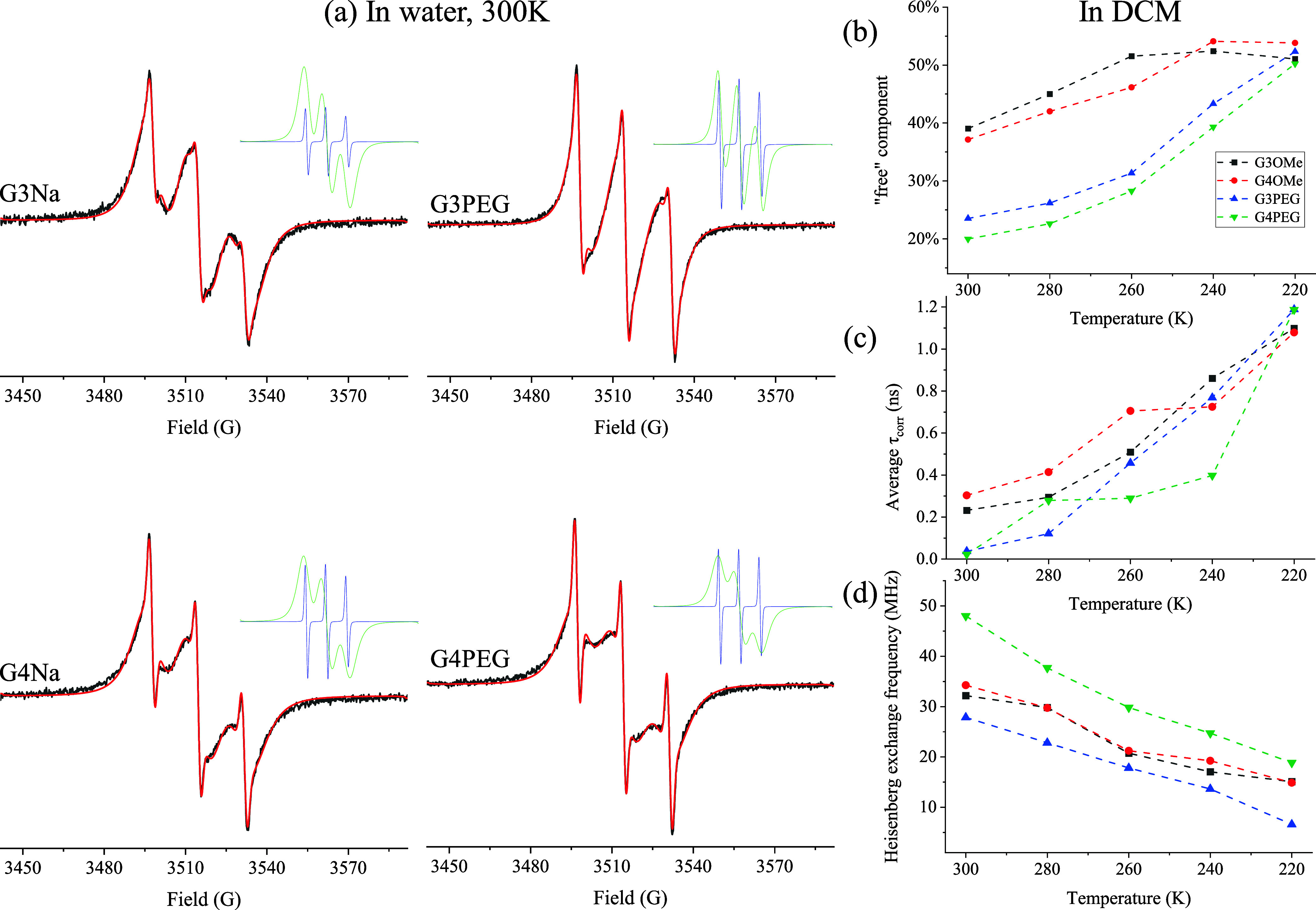
(a) EPR spectra of GnNa and GnPEG in water, at 0.63 mM per radical TEMPO unit, at 300 K, with experimental spectra shown in black and fitted spectra in red. In blue is represented the noninteracting “free” component, and in green is the exchange-coupled component. (b–d) Variable-temperature EPR spectra of GnOMe and GnPEG in DCM, at 0.5 mM per radical TEMPO unit, fitted from 300 to 220 K; (b) Percentage of the “free” component; (c) Average rotational correlation time (τ_corr_) of the two components; (d) Heisenberg exchange frequency (MHz) of the “exchange” component.

Subsequently, the experimental EPR spectra recorded in water at 300 K (Figure 5a) were simulated and analyzed using the EasySpin “chili” function. The simulated spectra showed excellent agreement with the experimental data, validating the chosen model. This analysis enabled the spectra to be decomposed into two components: the blue “free” component, corresponding to noninteracting radicals, and the green “exchange” component, representing radicals undergoing spin exchange interactions. The results (Table 1) indicate that, in both series, higher-generation dendrimers exhibit a greater contribution from the “exchange” component, stronger Heisenberg exchange frequencies, and slower rotational motion (higher τ_corr_). Furthermore, GnPEG dendrimers show faster radical mobility (lower τ_corr_) compared to GnNa, likely due to the reduced steric hindrance and greater flexibility provided by the PEG chains.^63^ This, in turn, leads to a slightly higher spin-exchange contribution.

**Table 1:** Parameters Obtained Using EasySpin from Spectra Recorded in Water at 300 K, with a TEMPO Radical Concentration of 0.63 mM

	“free” component		“exchange” component	
	τ_corr_ (ns)	contribution (%)	Heisenberg exchange freq (MHz)	contribution (%)
G3Na	0.31	39.04	25.89	60.96
G4Na	0.37	19.36	30.72	80.64
G3PEG	0.17	26.41	23.09	73.59
G4PEG	0.20	21.51	34.18	78.49

Variable-temperature EPR measurements were conducted for the GnOMe and GnPEG dendrimers in DCM over the range of 300 to 220 K. Across this temperature range, the spectra predominantly displayed the characteristic three-line pattern of nitroxide radicals (Figure S6). The experimental spectra were also simulated and analyzed using EasySpin. As the temperature decreased, the proportion of the “free” radical component gradually increased (Figure 5b), while the overall Heisenberg exchange frequency decreased (Figure 5d), indicating weaker dipolar and exchange interactions. Additionally, the rotational correlation time (τ_corr_) increased with decreasing temperature (Figure 5c), reflecting reduced radical mobility.

Overall, the radicals in GnPEG exhibited faster rotational dynamics (lower rotational correlation time) and slightly stronger radical interactions compared to the GnOMe series (Figure 5b,d), consistent with the trends observed in aqueous solution.

In summary, higher-generation dendrimers (G4) with more compact architectures restrict radical mobility and enhance radical–radical interactions. The observed temperature-dependent changes suggest that dendrimer structures become more rigid at lower temperatures, thereby limiting radical mobility and reducing the extent of their interactions. When comparing the different linkers, the GnPEG series exhibits greater flexibility than GnOMe, which results in both faster rotational dynamics (lower τ_corr_) and slightly enhanced spin–spin interactions. These data are essential for the subsequent analysis of relaxivity.

Accordingly, we proceeded to determine the relaxivity values for each system, as this parameter enables comparison of their effectiveness as contrast agents and helps identify if structural factors govern relaxivity.

### 3.4 MRI *In Vitro*

*T*_1_ *-*weighted MR images were acquired for aqueous solutions of GnNa and GnPEG dendrimers, and the resulting images are presented in Figure 6a. The images became brighter as the concentration of radical unit increased. Relaxivity is a parameter that evaluates the performance of contrast agents. Higher relaxivity means better contrast in the image. It has two components: *r*_1_ (longitudinal) and *r*_2_ (transverse). To measure the relaxivity of a contrast agent in solution, we plot the changes in relaxation rates (1/Δ*T*_1_) and (1/Δ*T*_2_) against the concentration. The slopes of the regression lines correspond to *r*_1_ and *r*_2_ (Figure 6b).

**Figure 6:**
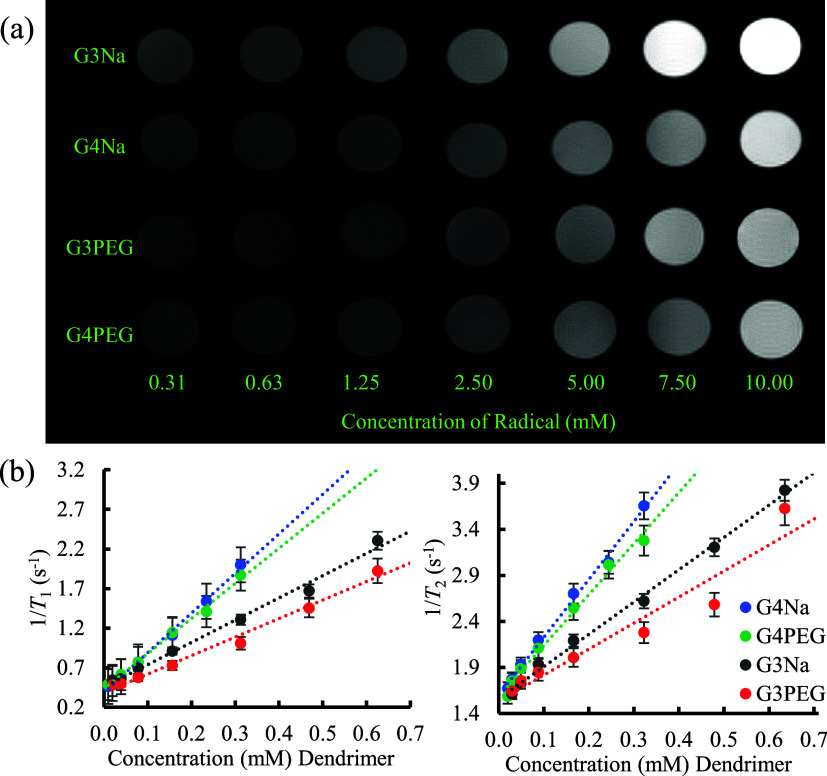
(a) *T*_1_-weighted images; (b) Plots of *r*_1_ (1/*T*_1_) and *r*_2_ (1/*T*_2_) versus concentration for dendrimer.

Table 2 presents the relaxivities of the dendrimers (Gn) per molecule in water at 300 K and 7 T, reported both per molecule and per TEMPO radical unit. For the entire dendrimer molecules, the *r*_1_ values of the fourth-generation compounds are higher than those of the third generation, with G4Na exhibiting a higher relaxivity than G4PEG (5.01 vs 4.42 mM^–1^ s^–1^). Notably, both G4Na and G4PEG surpass 4 mM^–1^ s^–1^, a value comparable to that of some clinically used GBCAs under similar conditions (7 T, water, 300 K), such as Gd-DTPA (3.25 mM^–1^ s^–1^) or Gd-DOTA (4.0 mM^–1^ s^–1^).^21^

**Table 2:** Relaxivity of Per Dendrimer and Per TEMPO Unit, in Water, 300 K, 7 T

	*r*_1_ (mM^–1^ s^–1^) per molecule	*r*_1_ (mM^–1^ s^–1^) per TEMPO	*r*_2_ (mM^–1^ s^–1^) per molecule	*r*_2_ (mM^–1^ s^–1^) per TEMPO	*r*_2_/*r*_1_
G2Na^49^	1.47	0.18	1.67	0.21	1.17
G3Na	2.81	0.18	3.51	0.22	1.25
G4Na	5.01	0.16	6.29	0.20	1.26
G3PEG	2.39	0.15	2.83	0.18	1.18
G4PEG	4.42	0.14	5.54	0.17	1.25
TEMPO	0.18	0.18	0.18	0.18	1.01
Gd-DTPA	3.25	**/**	3.79	**/**	1.17

Similarly, when considering *r*_1_ values per TEMPO unit, the GnNa series exhibits higher relaxivity than the GnPEG series and is comparable to free TEMPO (0.18 mM^–1^·s^–1^). In contrast, the GnPEG compounds show lower *r*_1_ per radical than both GnNa and free TEMPO. On the other hand, the third-generation dendrimers demonstrate marginally higher *r*_1_ per radical unit than their fourth-generation counterparts.

The higher the *r*_1_ value, the more effective the contrast agent is in enhancing the *T*_1_-weighted images^64^ while the higher the *r*_2_ value, the more likely the contrast agent is to cause signal loss and image distortion.^65^ Therefore, contrast agents with low *r*_2_/*r*_1_ values (less than 4 being optimal around 1) are considered good candidates for enhancing *T*_1_-weighted images, as they have minimal negative effects on the image quality,^66^ while *T*_2_ contrast agents exhibit *r*_2_/*r*_1_ ratios greater than 10.^67^ It can be seen from Table 2 that all compounds show *r*_2_/*r*_1_ equivalent to Gd-DTPA.

We analyzed the *r*_1_ relaxivity in relation to the rotational correlation time (τ_corr_) obtained from EPR measurements. Although τ_corr_ is higher for GnNa than for GnPEG, consistent with the higher *r*_1_ relaxivity observed in GnNa, this correlation does not hold across all compounds. Therefore, we sought to identify additional parameters influencing relaxivity.

Since all measurements were performed under identical experimental conditions, the effects of temperature and magnetic field strength on relaxivity can be considered negligible.^68^ For Gd^3+^ complexes, it is well established that increasing the effective hydration number and promoting a rapid exchange of water molecules with the metal center enhance *r*_1_.^69,70^ Similarly, improved water accessibility to TEMPO radicals may also contribute to higher relaxivity. These considerations led us to focus on the interface, particularly the interactions between water molecules and TEMPO units. To further investigate the interfacial mechanisms, we conducted molecular dynamics (MD) simulations on all four dendrimer compounds.

### 3.5 Molecular Dynamics Study of the Dendrimer–Water Interface

We constructed a molecular model for each radical dendrimer (Gn) and conducted 200 ns molecular dynamics (MD) simulations in an explicit water solvent (see Figure S7 and Table S1). Figure 7 depicts the molecular conformations after 200 ns. The root-mean-square deviation (RMSD, Figure 8a) indicates that the system reaches a relatively stable state after approximately 50 ns. Table 3 summarizes the radius of gyration (*R*_g_, Figure 8b), the aspect ratio (derived from the three principal moments of inertia: *I*_*x*_, *I*_*y*_, and *I*_*z*_), and the asphericity (δ) over the final 10 ns of simulation. It can be observed that *R*_g_ is greater for the fourth-generation molecules compared to the third generations, and higher-generation molecules exhibit shapes that are closer to spherical (lower asphericity), as evident in Figure 7.

**Figure 7:**
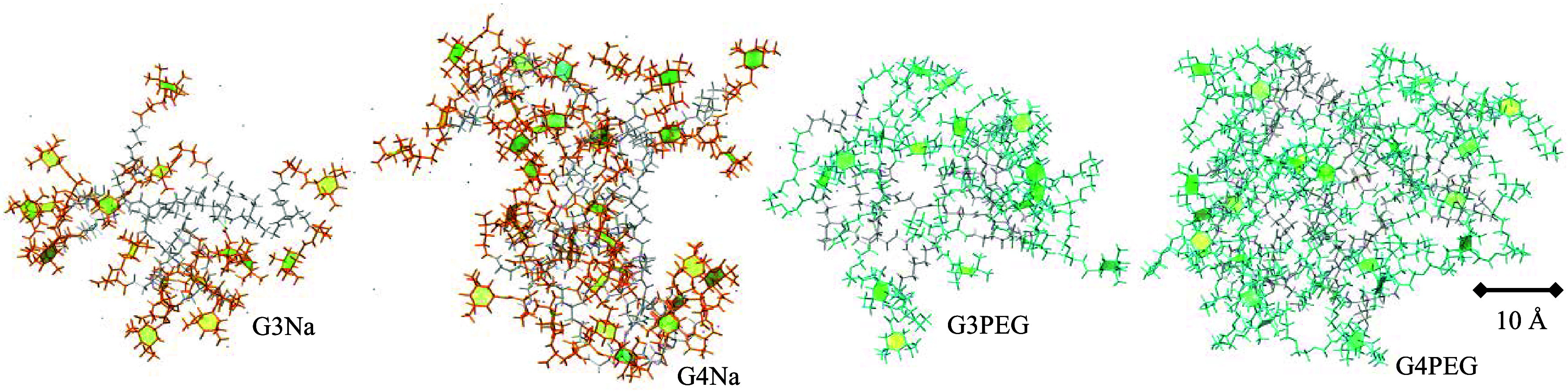
Dendrimer MD simulation snapshot at 200 ns. Polyamide dendrimer in gray, amino acid salt in orange, PEG in blue and TEMPO are filled in green.

**Figure 8:**
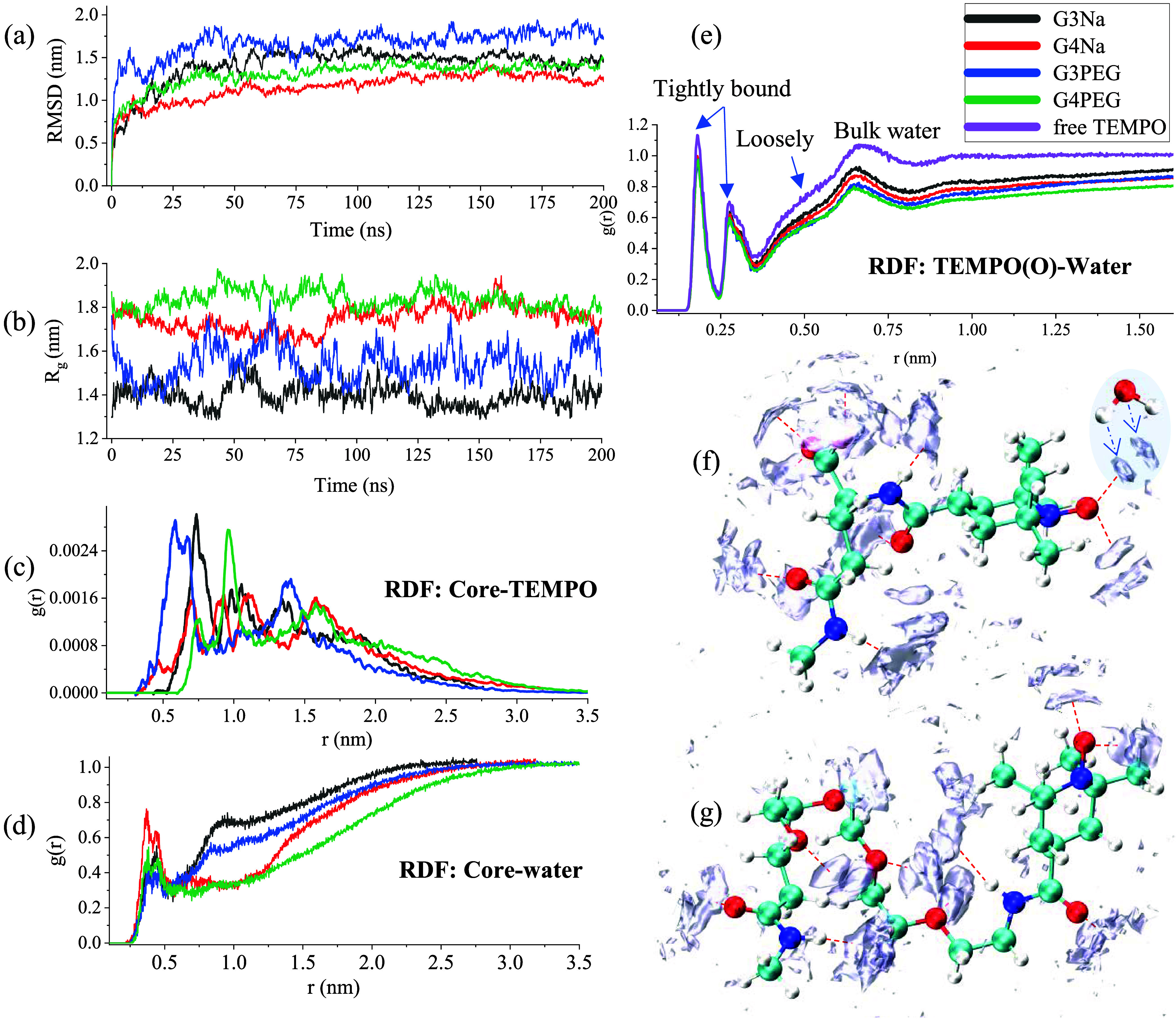
(a) RMSD of Gn; (b) *R*_g_ of Gn; (c) RDF of core-TEMPO­(O) of Gn; (d) RDF of core-water of Gn; (e) RDF of TEMPO­(O)-water of Gn; (f, g) SDF maps showing water (gray-blue) near the Glu-TEMPO branches (f) and PEG4-TEMPO branches (g), with hydrogen bonds indicated by red dotted lines.

**Table 3:** Information about the Molecular Structure of Dendrimers

			aspect ratios			H-bonds number	
	M.W. (g mol^–1^)	*R*_g_ (nm)	*I*_*x*_/*I*_*y*_	*I*_*x*_/*I*_*z*_	asphericities δ	TEMPO(O)	dendrimer
G3Na	7580.74	1.40	1.045	1.224	0.0036	1.73	224.17
G4Na	15,413.81	1.74	1.038	1.185	0.0025	1.71	457.31
G3PEG	9119.85	1.63	1.069	1.271	0.0049	1.67	198.80
G4PEG	18,492.06	1.78	1.061	1.187	0.0024	1.67	379.44

Figure 8c presents the radial distribution function (RDF) of the TEMPO oxygen atoms relative to the dendrimer core, illustrating the spatial arrangement of the radical units. The data reveal that TEMPO is not confined to the molecular periphery but undergoes significant “back-folding” toward the core, resembling the behavior of the amino termini in the precursor Gn-NH_2_,^57^ though TEMPO folds even deeper. Conversely, Figure 8d shows the water distribution, indicating a significantly lower water density within the dendrimer interior compared to the exterior interface. The juxtaposition of these two plots suggests that TEMPO radicals tend to bury themselves in a water-deficient environment, a phenomenon that likely limits their accessibility to water and, consequently, their potential relaxivity. Interestingly, G3Na, which has a lower atomic density, retains a relatively higher internal water content than the other dendrimers, potentially contributing to its superior *r*_1_ value.

To further probe the local hydration environment, spatial distribution function (SDF) maps were generated for the end-branched Glu-TEMPO and PEG4-TEMPO segments (Figure 8f,g). These maps show that water preferentially localizes near polar regions, specifically the TEMPO­(O) group, carboxylates, amides, and PEG chains, driven by hydrogen bonding capabilities (Figure S8), indicating that the amino acid-derived carboxylate sodium salt linkers facilitate the formation of a dense hydrogen-bonding network, whereas the PEG chains result in a more diffusive hydration interface. To quantify this effect, we calculated the number of hydrogen bonds (H-bonds) at the TEMPO­(O)–water interface (Table 3) and analyzed the RDF of surrounding water molecules (Figure 8e). The RDF profiles exhibit a distinct first hydration shell, representing tightly bound or “effective inner-sphere” water, followed by a second, loosely bound layer.

These computational data provide a robust mechanistic explanation for the observed relaxivity trends. Unlike Gd^3+^ (S = 7/2), where the strong magnetic moment allows for significant outer-sphere relaxation contributions,^69^ the relaxation efficiency of nitroxide radicals (S = 1/2) is inherently weaker and dominated by dipolar interactions with water protons in the immediate vicinity of the N–O^•^ moiety.^21^ Due to the steep distance dependence (Gd^3+^ is 1/*r* ^6^) and the weaker paramagnetic center, water molecules beyond this first hydration shell contribute negligibly to relaxation. Therefore, the effective relaxivity is strictly governed by the probability of finding water molecules within the hydrogen-bonding distance.

The number of H-bonds per radical in all dendrimers is lower than that of free TEMPO (avg. 1.95), reflecting the reduced hydration caused by back-folding and steric shielding. This explains why the per-radical *r*_1_ of the dendrimers does not exceed that of free TEMPO. Crucially, the GnNa series (particularly G3, Figure 8f) exhibits a higher probability of water occupancy near TEMPO­(O) and forms more H-bonds than the GnPEG series (Figure 8g). This enhanced local hydration creates a water-rich pocket that maximizes the dipolar interaction, directly correlating with the higher experimental *r*_1_ values observed for the amino acid-linked dendrimers.

In summary, the relaxivity of these dendrimers is governed by a balance between the inward folding of radicals into the hydrophobic core (which reduces water access) and the ability of the linker to organize interfacial water (which promotes hydrogen bonding). These findings highlight that maximizing the relaxivity of metal-free agents requires the simultaneous optimization of radical positioning to prevent burial and linker chemistry to enhance local hydration density.

### 3.6 Cytotoxicity

*In vitro* cell viability assays were performed to evaluate the cytotoxicity of G3-G4Na and G3-G4PEG using the African green monkey kidney (Vero) cell line. Cells were incubated with varying concentrations of dendrimer (2 mM to 0.0625 mM per radical unit) for 24 h, and viability was assessed using the resazurin assay. All four species demonstrated negligible cytotoxicity, as indicated by consistently high cell viability (Figure S9). Based on these results, *in vivo* studies in mice were subsequently initiated.

### 3.7 MRI Study of Pharmacokinetics and Glioblastoma Diagnosis in Mice

Given the higher *r*_1_ relaxivity and superior water solubility of G4Na compared to G4PEG, G4Na was selected as the best candidate for *in vivo* studies, including pharmacokinetics and tumor imaging performance.

The pharmacokinetics of G4Na radical dendrimers was evaluated in healthy mice using dynamic contrast-enhanced MRI (DCE-MRI). *T*_1_-weighted MR images were acquired over a 17 min period following intravenous administration of G4Na (0.072 mmol/kg per dendrimer) to track its biodistribution and clearance pathways. As shown in Figure 9a, immediately after injection, a pronounced increase in the relative enhancement of signal intensity was observed in the renal cortex and pelvis, indicating rapid filtration of G4Na via the kidneys. The relative enhancement of signal intensity in the renal cortex reached its maximum approximately 8 min postinjection, followed by a gradual decline, suggesting effective glomerular filtration and subsequent clearance. In contrast, an increase over time in the relative enhancement of signal intensity in the bladder was evident from around 5 min postinjection, steadily increasing throughout the experimental period. This pattern confirmed that G4Na was efficiently excreted through renal pathways.

**Figure 9:**
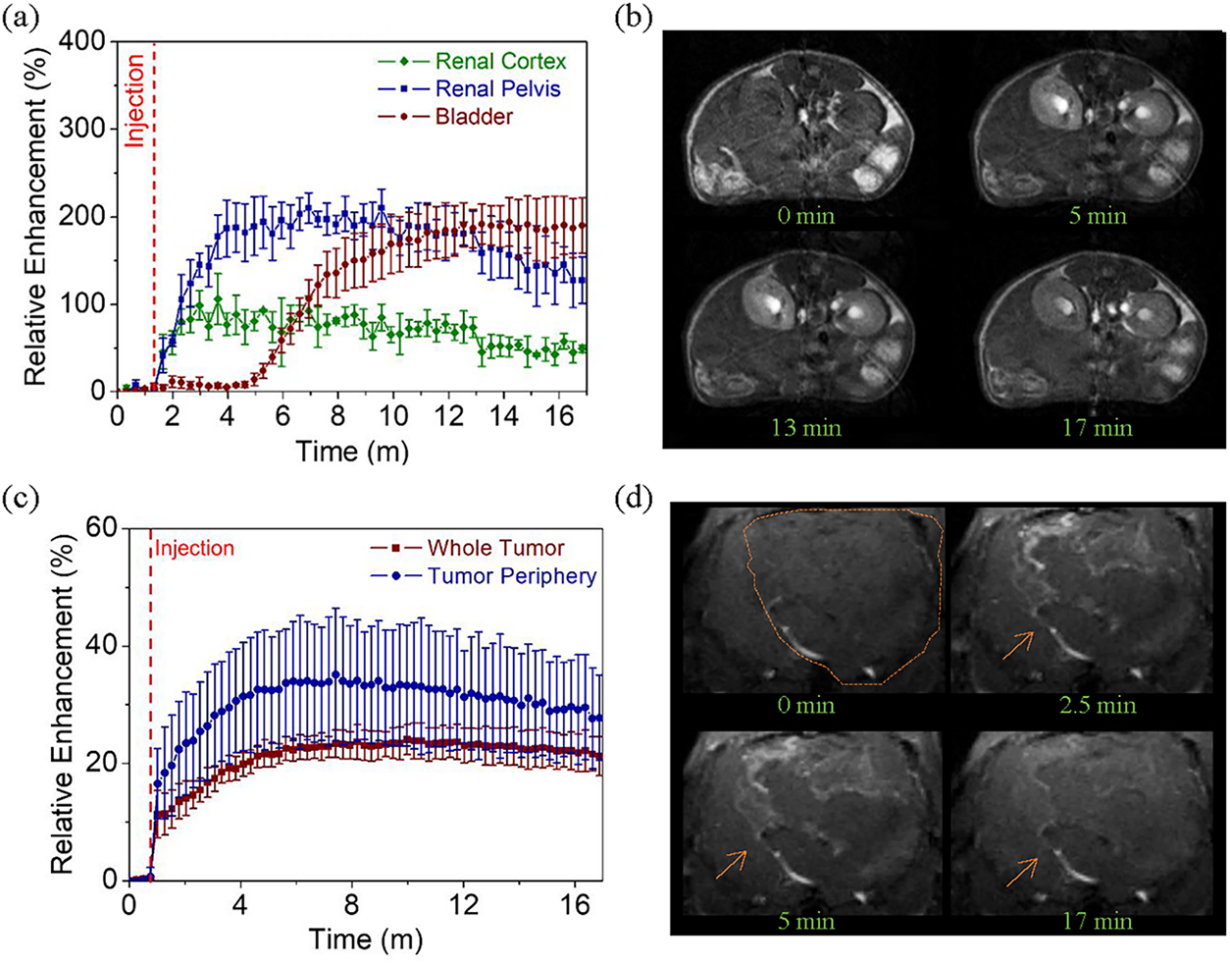
(a) *In vivo* pharmacokinetics (DCE-MRI) of renal cortex, renal pelvis, and bladder after the intravenous administration of G4Na, in mice; (b) Representative *T*_1_-weighted *MR* images from the DCE-MRI sequence at different experimental time points of kidneys; (c) *In vivo* pharmacokinetics (DCE-MRI) of whole tumor (red) and tumor periphery (blue) after the intravenous administration of G4Na; (d) Representative *T*_1_-weighted brain *MR* images (axial view) from the DCE-MRI sequence at different experimental time points showing contrast enhancement in the glioblastoma periphery (these images correspond to T_2_-weighted images in Figure S11). The arrows indicate the tumor location and the characteristic ring-like enhancement at the tumor periphery.

Taking into account the size of G4Na (*R*_g_ 1.74 nm, i.e., diameter 3.48 nm, below 10 nm), this facilitates its filtration through the glomerular capillaries, similar to other small-molecule contrast agents.^71,72^ Importantly, no significant signal changes were detected in the liver or spleen, indicating minimal off-target accumulation and ensuring a high degree of specificity for renal clearance. *T*_1_-weighted MR images captured at different time points further illustrated the observed pharmacokinetic behavior (Figure 9b). Therefore, the rapid renal clearance and lack of off-target accumulation highlight its favorable pharmacokinetic profile, reducing the risk of prolonged retention and systemic toxicity.

Following DCE-MRI, mice were euthanized, and the liver and kidneys were evaluated to identify acute damage (25 min after administration), as subacute or prolonged damage was not within the scope of this work. At the tissue level, G4Na did not induce any apparent morphological changes, as the liver and kidney structures remained comparable to those observed in the control group (injected with 0.9% NaCl) (Figure S10).

DCE-MRI is also a highly effective technique for cancer diagnosis, particularly in the assessment of high-grade gliomas. The characteristic DCE-MRI profile of human high-grade gliomas includes a rapid and intense contrast uptake at the tumor periphery, corresponding to highly vascularized proliferative regions and a slower accumulation in the tumor core, indicative of necrotic areas.^73^ In proliferative regions, G4Na begins to wash out within minutes, further confirming the high vascularization of these areas. This pattern reflects the underlying vascular pathology and the disruption of the blood–brain barrier (BBB) associated with these aggressive brain tumors, leading to the formation of a pathological interface known as the blood–brain tumor barrier (BBTB).^74^ Following the intravenous administration of G4Na, a rapid signal enhancement was observed, consistent with the expected extravasation of small molecules (hydrodynamic diameter <10 nm) into the tumor interstitium (Figure 9c). This initial signal increase was followed by a gradual signal decay, corresponding to the clearance of the G4Na from the tumor. Importantly, no significant differences were observed between the whole tumor and the tumor periphery in this experiment, except for a moderate earlier onset of washout in the tumor periphery. Remarkably, this behavior closely resembled that of commercial GBCAs, such as Gadovist (Bayer AG, Leverkusen, Germany), which also exhibits a hydrodynamic diameter below the renal exclusion limit. A previous study by some of us with Gadovist demonstrated similar pharmacokinetics in a rat glioblastoma model developed using the same cancer cells,^75^ highlighting the potential of G4Na as a promising CA for DCE-MRI applications. *T*_1_-weighted MR images further illustrated these findings, revealing a pronounced signal enhancement at the tumor periphery 1.5 min postinjection (2.5 min after the beginning of the experiment), which became more pronounced at 4 min postinjection, followed by a gradual signal reduction toward the end of the experiment (Figure 9d). The corresponding *T*_2_-weighted images are depicted in Figure S11 for better visualization of the tumor mass. In addition, EPR analysis of brain tumor tissue collected from mice 25 min after intravenous administration of G4Na revealed a clear EPR signal corresponding to the radical dendrimer in the tumor regions (Figure S12).

## 4 Conclusions

In this study, we synthesized radical dendrimers based on polyamide scaffolds of generations G3 and G4, comprising 16 and 32 terminal branches, respectively. Two distinct strategies were employed to render these dendrimers water-soluble while preserving their capacity for full functionalization with organic radicals—one radical per terminal branch. These strategies involved the use of (i) amino acid-derived carboxylate sodium salts and (ii) PEG chains as linkers between branches and radicals. Unlike conventional approaches that rely on statistical functionalization, our two approaches allow precise control over both the dendrimer structure and the number of radicals introduced, ensuring reproducibility in the synthesis. Optimization of PEG length identified PEG4 as the ideal compromise, providing sufficient water solubility, synthetic accessibility, and complete radical functionalization.

*In vitro* MRI measurements demonstrated strong *T*_1_ contrast enhancement, with GnNa dendrimers exhibiting higher *r*_1_ relaxivity values than their GnPEG counterparts. The highest *r*_1_ was recorded for G4Na, reaching 5.01 mM^–1^ s^–1^, a value exceeding those of commercial GBCAs.

When normalized per radical unit, the trend in *r*_1_ followed: G3Na > G4Na > G3PEG > G4PEG. Although τ_corr_ (extracted from EPR simulations) was higher for GnNa than for GnPEG—consistent with the higher *r*_1_ values of GnNa—this correlation was not consistent across all compounds, suggesting that additional factors contribute to the observed relaxivity differences.

To elucidate these factors, molecular dynamics simulations were performed to probe the dendrimer–water interfacial behavior. Three primary determinants of *r*_1_ relaxivity were identified: the spatial distribution of TEMPO radicals within the dendrimer architecture, the distribution of water molecules, and the extent of hydrogen bonding between the radicals and surrounding water molecules at the interface. The results confirmed that relaxivity is positively correlated with radical accessibility to water and the formation of stable hydration shells at the interface—underscoring the importance of dendrimer design in optimizing MRI contrast performance.

G4Na was selected for *in vivo* MRI studies due to its highest *r*_1_ relaxivity among the synthesized dendrimers and its superior water solubility. *In vivo* pharmacokinetic analysis revealed that G4Na undergoes rapid renal clearance with minimal off-target accumulation, exhibiting a clearance profile comparable to that of clinically used gadolinium-based contrast agents. In addition, G4Na exhibited no cytotoxicity and no signs of *in vivo* toxicity, with no evidence of acute tissue damage. Remarkably, G4Na demonstrated strong performance in glioblastoma tumor imaging, showing pronounced signal enhancement at the tumor periphery. This enhancement is consistent with regions of increased vascularization and compromised blood–brain barrier integrity, typical of glioblastoma pathology.

Overall, this work demonstrates that the amino acid–based carboxylate linker outperformed PEG in both solubility and relaxivity, supporting its potential as the preferred design strategy for future dendrimer-based contrast agents.

## Supplementary Material



## Data Availability

Data will be made available on request.

## Notes

Synthetic procedures and characterization (FT-IR, ^1^H NMR, EPR) of all intermediates and final dendrimers; detailed parameters for molecular dynamics simulations; cell viability assay data; histological analysis of tissues; and *in vivo* MRI experimental details including normalization methods (PDF)
