# Supplementary material for: Designing Water-Soluble Macromolecules for Biomedical Use: PEG Chains versus Amino Acids—A Case Study in MRI Contrast Agents
Source: ACS Polym Au. 2026 Feb 17;6(3):806–20. doi: 10.1021/acspolymersau.5c00162 (PMC13261739; doi:10.1021/acspolymersau.5c00162)
Supplement: Supplementary file 1 [file lg5c00162_si_001.pdf]

Supporting Information (SI)

Designing Water-Soluble Macromolecules for Biomedical Use: PEG Chains versus Amino Acids. A Case Study in MRI Contrast Agents.

Yufei Wu, [a] Carlos Caro,[b,c] Esther Matamoros,[d,c] Jesús David Urbano-Gámez,[b,c] Silvia Lope-Piedrafita,[e,f] Yolanda Vida,[d,c] María Luisa García-Martín,[b,c,g] José Vidal-Gancedo,[a,f] Vega Lloveras [a,f *]

[a] *Institut de Ciència de Materials de Barcelona, ICMAB-CSIC, Campus UAB, 08193 Bellaterra, Spain*
[b] *Biomedical Magnetic Resonance Laboratory-BMRL, Andalusian Public Foundation Progress and Health-FPS, 41092 Seville, Spain*
[c] *Instituto de Investigación Biomédica de Málaga y Plataforma en Nanomedicina−IBIMA, Plataforma Bionand, ParqueTecnológico de Andalucía, 29590 Málaga, Spain*
[d] *Departamento de Química Orgánica,Universidad de Málaga, 29071 Málaga, Spain.*
[e] *Departament de Bioquímica i Biologia Molecular, Unitat de Biofísica, Facultat de Medicina, Universitat Autònoma de Barcelona, 08193 Bellaterra, Spain*
[f] *Networking Research Center on Bioengineering, Biomaterials and Nanomedicine, CIBER-BBN, Instituto de Salud Carlos III, Campus UAB, 08913 Bellaterra, Spain*
[g] *Networking Research Center on Bioengineering, Biomaterials and Nanomedicine, CIBER-BBN, 29590 Málaga, Spain*
[*] *Corresponding author*
E-mail: vega@icmab.es (V.L.M.)

1. Synthesis

1.1. *General procedure for amidation reaction: Gn-NH$_2$ with Linker-COOH* ***(i)***

In a 5 mL schlenk bottom flask, under Nitrogen (N_2) atmosphere, BocNH-(PEG)4-COOH (1.2 eq) or Boc-Glu-OMe-COOH (1.2 eq) and HATU (1.2 eq) were dissolved in anhydrous DCM, followed by the addition of *N*,*N*-Diisopropylethylamine (DIEA, 27 eq) and the mixture was stirred for 1h. After that, G3-NH$_2$-16HCl (1/16 eq) or G4-NH$_2$-32HCl (1/32 eq) were slowly added and the mixture was stirred at room temperature for 48 hours.

The organic layer was washed with 0.02 M $NaHCO_3$ (2×10 mL), brine (3×10 mL) and dried over anhydrous $MgSO_4$. Then, the solution was filtered and precipitated into *n*-hexane (DCM/*n*-hexane < 1/5, 2×10mL), Ether (DCM/Ether < 1/5, 2×10mL). Following centrifugation and drying, both compounds were obtained in yields exceeding 75%.

1.2. General procedure for tert-Butyl Carbamate (Boc) deprotection ***(ii)***

To deprotect the Boc group, first we dissolved 50 mg of the product from step **(i)** in 1 mL of DCM. Then, 1 mL of trifluoroacetic acid (TFA) was slowly added to the solution, which was then stirred at room temperature for 16 hours. The resulting mixture was then precipitated into cold *n*-hexane (1×10 mL), dissolved in 1 mL of methanol, and re-precipitated into ether (3×10 mL). The precipitated product was then centrifuged and dried, yielding a white compound (-$NH_4^+TFA^-$ salt) with a yield greater than 95%.

1.3. *General procedure for amidation reaction: Gn-Linker-NH$_2$ with TEMPO-COOH* ***(iii)***

In a 5 mL Schlenk bottom flask, under Nitrogen (N_2) atmosphere, covered with aluminum foil, TEMPO-COOH (1.2 eq) and HATU (1.2 eq) were dissolved in anhydrous DCM. After that, DIEA (27 eq) was added and the mixture was stirred for 1h. Then, G3-NH$_2$-16TFA (1/16 eq) or G4-NH$_2$-32TFA (1/32 eq) from **(ii)** were added to the mixture slowly and it was stirred at room temperature for 48 hours.

The organic layer was washed with 0.02 M $NaHCO_3$ (2×10 mL) and brine (3×10 mL), then dried over anhydrous $MgSO_4$, and finally, the solution was filtered and precipitated into *n*-hexane (DCM/n-hexane < 1/5, 2×10mL) and/or Ether (DCM/Ether < 1/5, 2×10mL). After centrifugation and drying, the yield of both reactions was higher than 85%.

1.4. General procedure for synthesis of dendrimer salts ***(iv)***

50 mg of G3-OMe-16TEMPO or G4-OMe-16TEMPO dendrimers were dissolved in a mixture of H_2O/THF (2 mL/1 mL) and it was stirred overnight with 20 eq of NaOH. Then, the solution was dialyzed for one day, changing ultrapure water every 2, 4, 6 and 12 h. Finally, the solution was freeze-dryed for three days to obtain the product.

2. Structure Characterization

2.1. IR

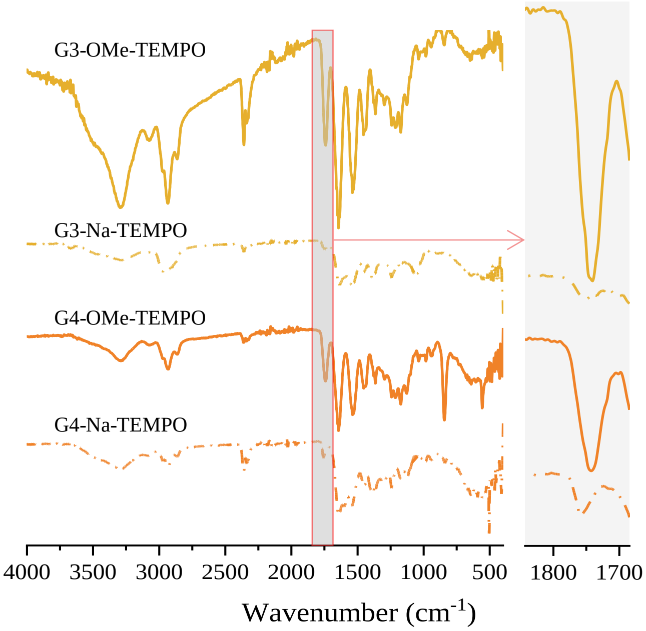

Figure S1. IR spectra of ester groups converted into carboxylate groups.

2.2. ^{1}H NMR

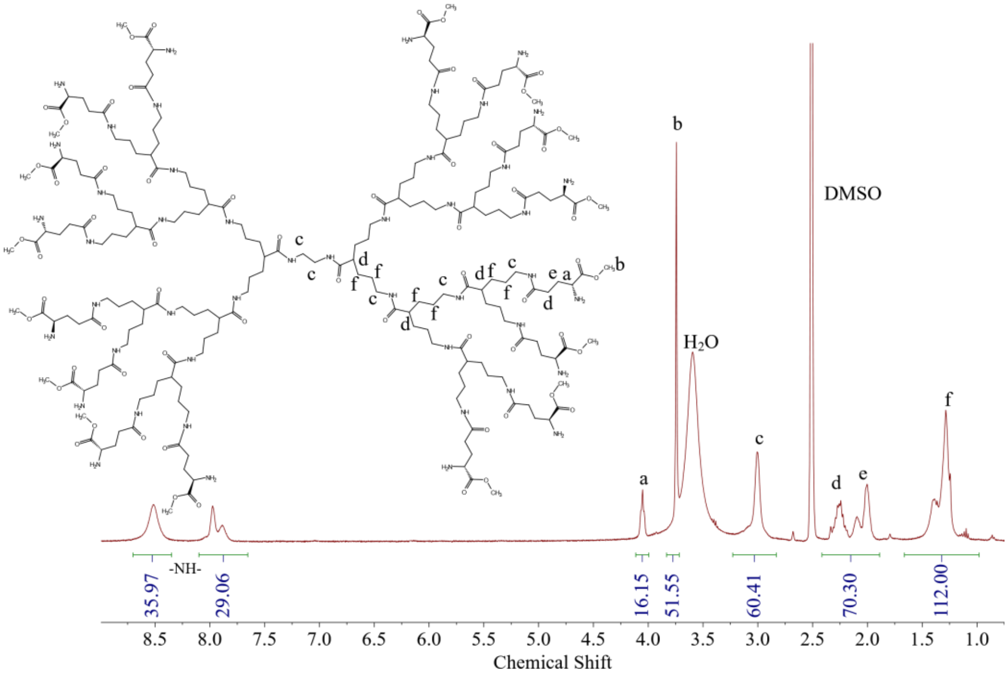

Figure S2. G3-16OMe-16NH$_2$ with TFA, DMSO-d$_6$.

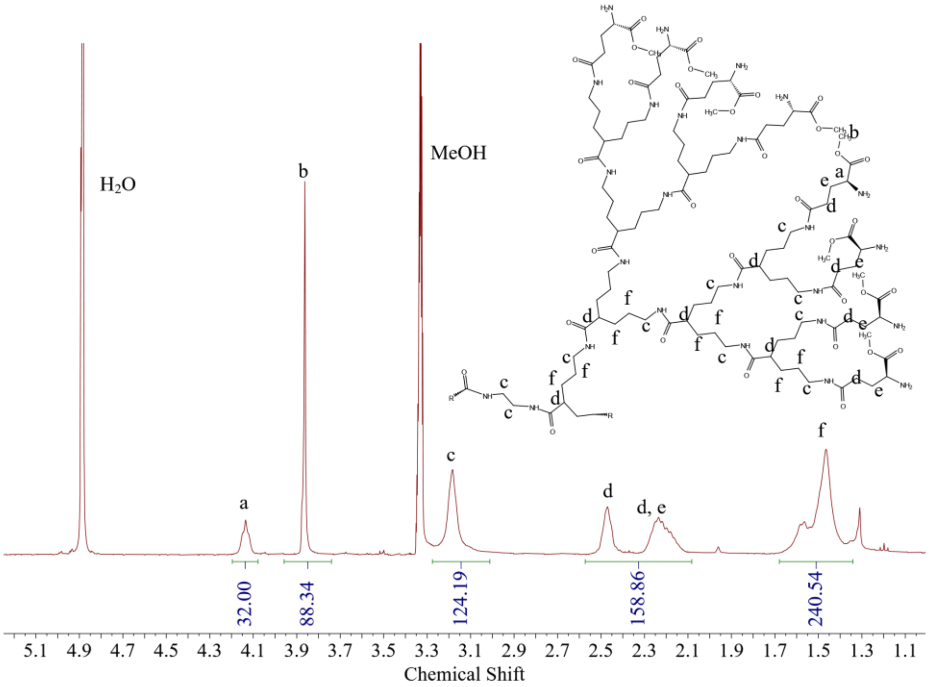

Figure S3. G4-32OMe-32NH$_2$ with TFA, MeOD.

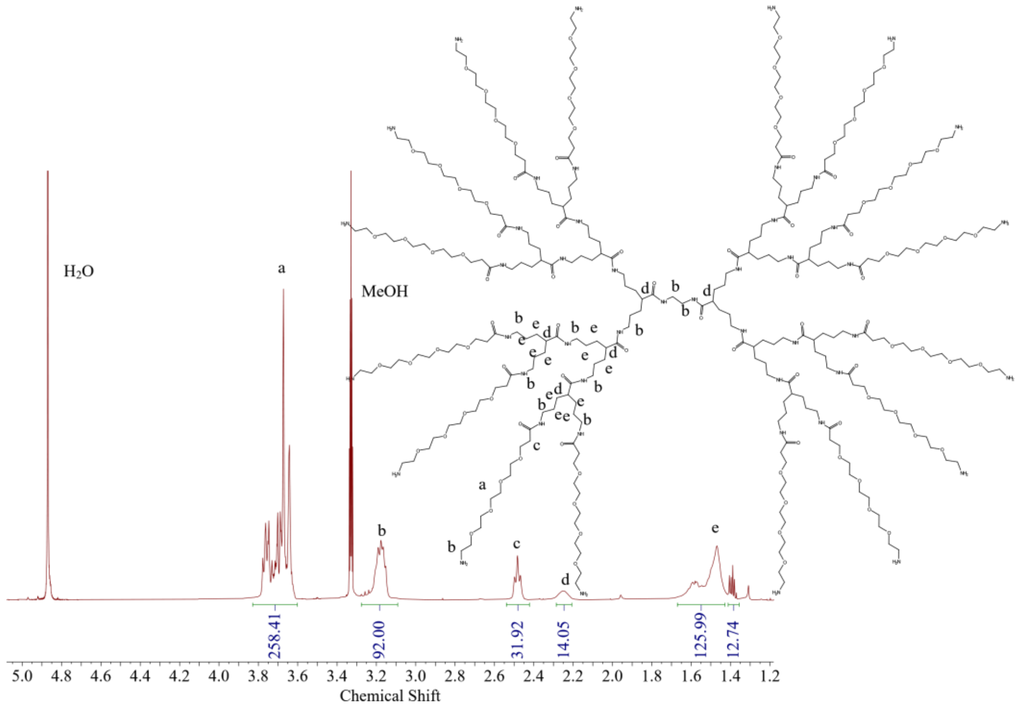

Figure S4. G3-16PEG4-16NH$_2$ with TFA, MeOD.

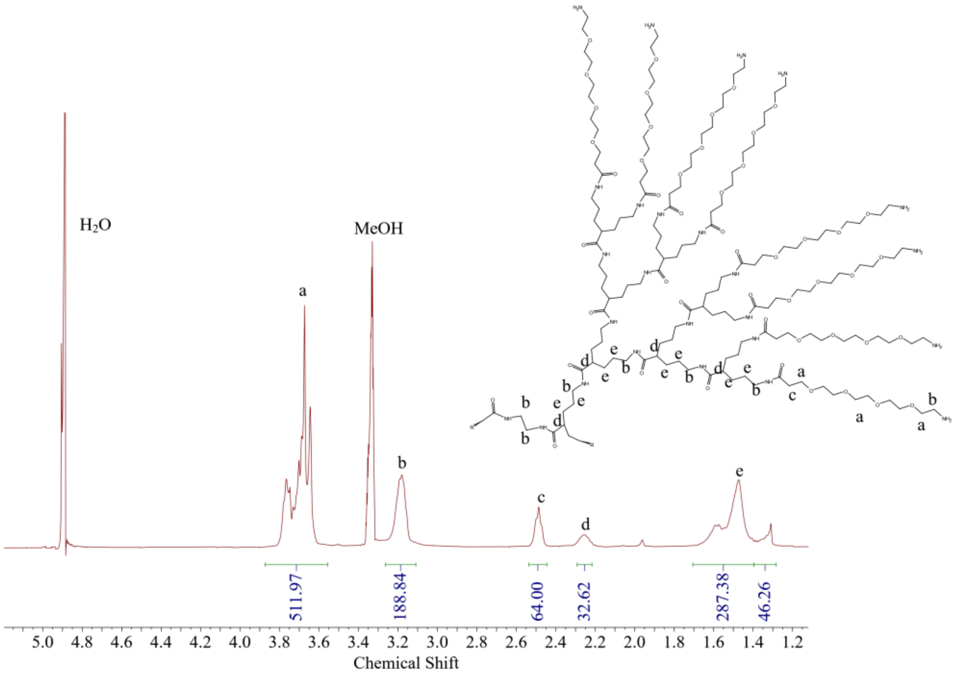

Figure S5. G4-32PEG4-32NH$_2$ with TFA, MeOD.

3. EPR

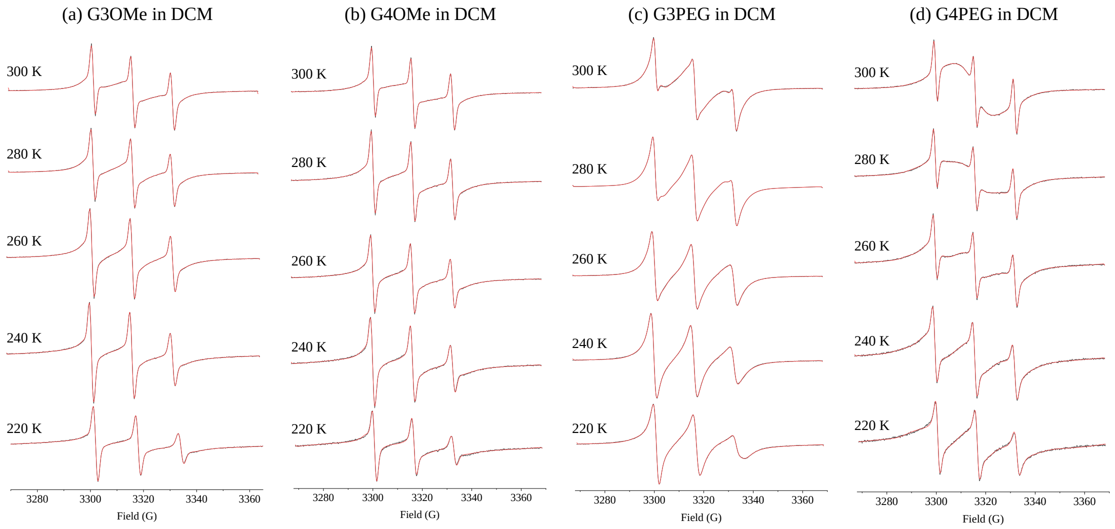

Figure S6. Variable temperature EPR spectra in DCM of G3/G4OMe and G3/G4PEG in DCM at 0.5mM per radical unit, with experimental spectra shown in black, and fitted spectra in red.

4. MD

The dendrimers were built based on three capped-residues: aaa (core), bbb (repetitive units), ccc (terminal unit) (**Figure S7**). The end group is protected by a cap, which will eventually be removed. It was used Dendrimer Builder Toolkit (DBT).[1]

All molecules were built up using Chemdraw and GaussView 6.[2] Molecules were pre-optimized using the GFN-2 general force field of the XTB program.[3,4] Partial charges were calculated by the restrained electrostatic potential (RESP) fitting method at the b3lyp/6-31g(d) level, contains the implicit solvent model scrf = smd, using Gaussian 16.[5,6]

Multiwfn was used to constrain the atomic charge.[7] The "cap" charge was set to 0, the carboxyl structure charge remained -1, the "cap" was removed, and DBT was used to build the molecule.

AmberTools 22 MD software was used to create a water box.[8] The General Amber Force Field (GAFF) has been employed for force field parameters.[9] It was used the TIP3P water model, in a tetrahedron cell.[10] In all simulations, a solvation shell of at least 10 Å was maintained around the dendrimer structure. ACPYPE (AnteChamber PYthon Parser interfacE) was used to convert topology files from AmberTools 22, into a format compatible with Gromacs.[11]

Gromacs version 2019.2 was used to perform all-atom MD simulations in explicit solvent.[12]

All simulations were conducted using 3D-periodic boundary conditions, and long-range electrostatics were accounted for by implementing the particle-particle mesh Ewald (PME) summation method. The simulations were initiated with random velocities at a temperature of 300 K. To maintain the temperature, a V-rescale thermostat with a coupling time of 0.1 ps was used, while the Parrinello–Rahman barostat with a coupling constant of 2 ps controlled the pressure. Integration of the Newton equations of motion was performed using the leap-frog algorithm with a time step of 1 fs. The LINCS algorithm was utilized to restrain the bonds involving hydrogens. Prior to production runs, each system underwent energy minimization using the steepest descent algorithm for 5000 steps. The equilibration of each system was performed at a temperature of 300 K for 100 ps in the NVT ensemble, followed by an additional equilibration step in the NPT ensemble with a reference pressure of 1 bar and temperature of 300 K for 100 ps. During the simulation,

the integration time step was set to 2 fs, production runs of 200 ns were carried out. Representative frames were taken in the last 1 ns for structural analysis.

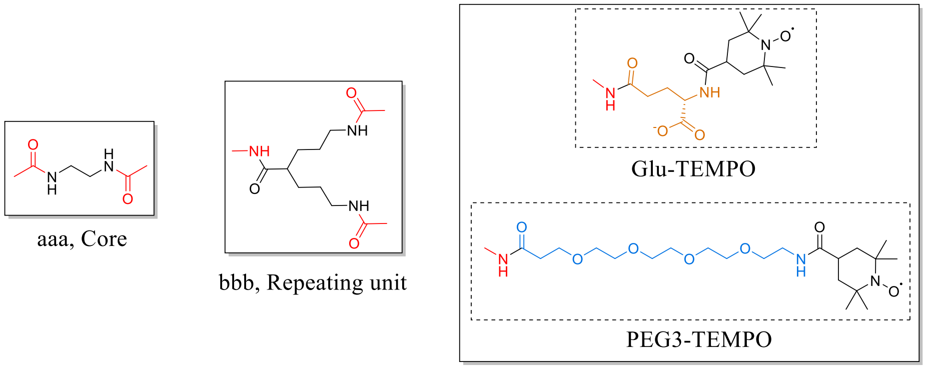

Figure S7. Residues selection for the dendrimers, Cap atoms are in red.

Table S1. Initial properties of dendrimers and simulation details.

	M.W. ($g\ mol^{-1}$)	N Tempo	N Dendrimer	N Na^+	N water	N Atoms	V_{box} (nm^3)
G3Na	7580.74	16	1110	16	8167	25611	255.83
G4Na	15413.81	32	2262	32	13590	43032	431.84
G3PEG	9119.85	16	1462	0	20020	61522	619.69
G4PEG	18492.06	32	2966	0	31941	98789	996.42

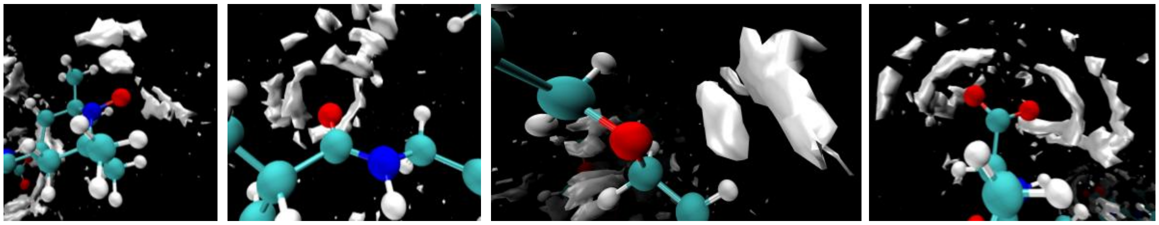

Figure S8. Differences in water distribution around different functional groups.

5. Cell viability assay

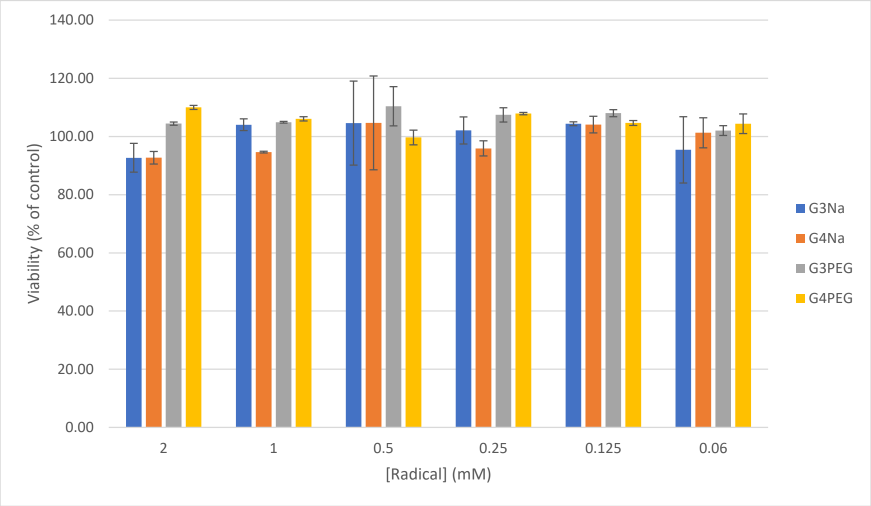

Figure S9. Resazurin cell viability assay of Vero cells with radical dendrimers G3-G4Na and G3-G4PEG at different concentration per radical unit. Blank: DMEM+1% FBS (without cells), negative control: DMEM+1% FBS cell culture media, and positive control: DMSO 20% in DMEM+1% FBS.

6. Animal Housing and Care

Mice were housed in individually ventilated cages (IVCs) under controlled environmental conditions: temperature of 21 ± 2°C, relative humidity of 55 ± 10% and a 12-hour light/dark cycle (lights on at 08:00). Up to 5 animals were housed per cage, depending on the experimental protocol and social compatibility.

Animals had ad libitum access to standard laboratory chow and filtered tap water. Bedding was changed twice weekly and environmental enrichment (e.g., nesting material, cardboard shelters) was provided throughout the experiment to promote well-being and natural behavior. All animals were acclimatized for at least 7 days prior to experimentation. Health status was monitored daily by trained personnel and humane endpoints were applied in accordance with ethical standards.

7. Histology

Histology. The tissues (liver and kidneys) were fixed in 4% formaldehyde (Panreac, pH 7 buffered) for 48 h, changing the 4% formaldehyde after 24 h. Then, the samples were dehydrated through graded ethanol, and embedded in paraffin (temperature 56 °C for 2 h under stirring and vacuum). The detailed procedures are described below.

Haematoxylin and Eosin (H&E): paraffin-embedded samples were sectioned at 7 μm thickness, then deparaffinized, rehydrated and stained with H&E. After that, samples were dehydrated in ascending concentrations of ethanol, cleared in xylene, and mounted on commercial glass slides.

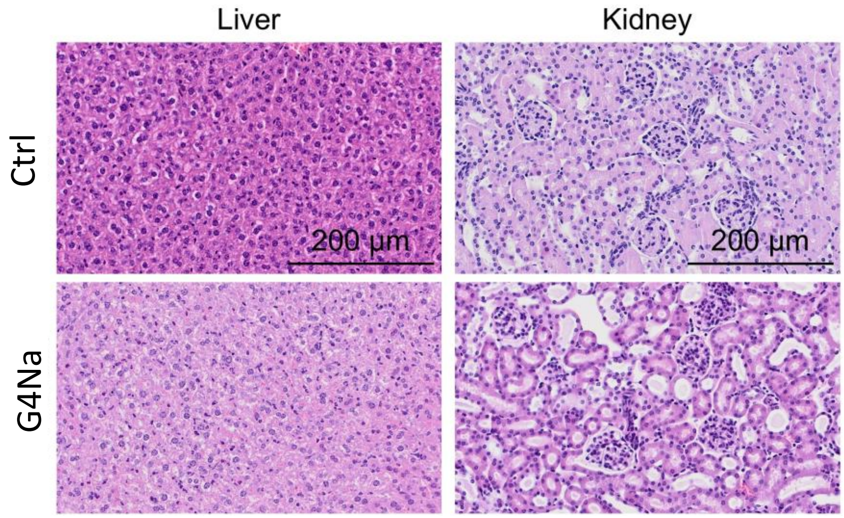

Figure S10. Histological analysis of liver and kidney tissues from mice after DCE-MRI, comparing intravenous administration of G4Na (bottom panels) with 0.9% NaCl saline injection as control (top panels).

8. MRI *in vivo*

Biodistribution and Pharmacokinetics Experiments: A 40 mm quadrature birdcage resonator was employed for signal acquisition.

T_1 Dynamic Contrast-Enhanced MRI (DCE-MRI) was performed using a turbo-RARE sequence (TE = 6 ms, TR = 550 ms, 1 average, 52 repetitions, FOV = 32x32, matrix size = 192x192, slice thickness = 1 mm).

Tumor Targeting Experiments in Glioma-Bearing Mice: Excitation was performed using a 72 mm volume resonator, while signal reception was achieved via a 10 mm inner-diameter surface coil positioned over the mice's heads. Tumor growth was monitored until reaching a volume of 15–20 mm^3, as determined by volumetric MRI.

DCE-MRI was acquired using a FLASH sequence (TE = 2.5 ms, TR = 40 ms, 2 averages, 70 repetitions, flip angle = 40º, matrix size = 192x192, FOV = 20x20 mm, slice thickness = 0.8 mm).

The experimental protocol followed the same steps in both experiments: (i) animal cannulation, (ii) positioning within the magnet, and (iii) DCE-MRI sequence.

Pharmacokinetics were obtained by calculating the average values within different regions of interest (ROIs) on the tissues.

Analysis: DCE-MRI was analyzed semiquantitatively using the following expression:

$$RE = \left|\frac{I_t - I_0}{I_0} \times 100\right|$$

where RE is the modulus of relative signal enhancement, I_t is the signal intensity at any given time after the injection, and I_0 is the signal intensity before the injection.

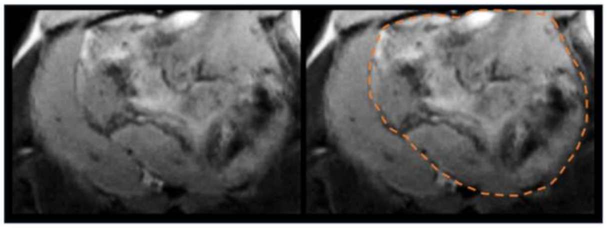

Figure S11. T_2-weighted *MR* images displaying the glioblastoma on an axial slice (outlined on the right image).

9. EPR of the brain tumor after intravenous administration of G4Na

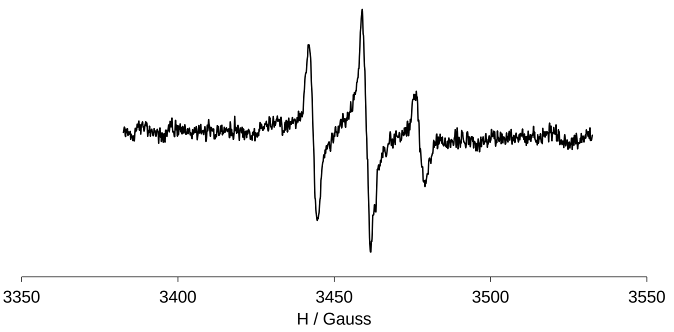

Figure S12. EPR spectrum of the brain tumor after intravenous administration of G4Na.

References

(1) Maingi, V.; Jain, V.; Bharatam, P. V.; Maiti, P. K. Dendrimer Building Toolkit: Model Building and Characterization of Various Dendrimer Architectures. *J. Comput. Chem.* **2012**, *33* (25), 1997–2011. https://doi.org/10.1002/jcc.23031.

(2) Dennington, R.; Keith, T. A.; Millam, J. M. GaussView Version 6, 2019.

(3) Bannwarth, C.; Caldeweyher, E.; Ehlert, S.; Hansen, A.; Pracht, P.; Seibert, J.; Spicher, S.; Grimme, S. Extended Tight-Binding Quantum Chemistry Methods. *WIREs Comput. Mol. Sci.* **2021**, *11* (2), e1493. https://doi.org/10.1002/wcms.1493.

(4) Spicher, S.; Grimme, S. Robust Atomistic Modeling of Materials, Organometallic, and Biochemical Systems. *Angew. Chem. Int. Ed.* **2020**, *59* (36), 15665–15673. https://doi.org/10.1002/anie.202004239.

(5) Bayly, C. I.; Cieplak, P.; Cornell, W.; Kollman, P. A. A Well-Behaved Electrostatic Potential Based Method Using Charge Restraints for Deriving Atomic Charges: The RESP Model. *J. Phys. Chem.* **1993**, *97* (40), 10269–10280. https://doi.org/10.1021/j100142a004.

(6) Frisch, M. J.; Trucks, G. W.; Schlegel, H. B.; Scuseria, G. E.; Robb, M. A.; Cheeseman, J. R.; Scalmani, G.; Barone, V.; Petersson, G. A.; Nakatsuji, H.; Li, X.; Caricato, M.; Marenich, A. V.; Bloino, J.; Janesko, B. G.; Gomperts, R.; Mennucci, B.; Hratchian, H. P.; Ortiz, J. V.; Izmaylov, A. F.; Sonnenberg, J. L.; Williams-Young, D.; Ding, F.; Lipparini, F.; Egidi, F.; Goings, J.; Peng, B.; Petrone, A.; Henderson, T.; Ranasinghe, D.; Zakrzewski, V. G.; Gao, J.; Rega, N.; Zheng, G.; Liang, W.; Hada, M.; Ehara, M.; Toyota, K.; Fukuda, R.; Hasegawa, J.; Ishida, M.; Nakajima, T.; Honda, Y.; Kitao, O.; Nakai, H.; Vreven, T.; Throssell, K.; Montgomery, J. A., Jr.; Peralta, J. E.; Ogliaro, F.; Bearpark, M. J.; Heyd, J. J.; Brothers, E. N.; Kudin, K. N.; Staroverov, V. N.; Keith, T. A.; Kobayashi, R.; Normand, J.; Raghavachari, K.; Rendell, A. P.; Burant, J. C.; Iyengar, S. S.; Tomasi, J.; Cossi, M.; Millam, J. M.; Klene, M.; Adamo, C.; Cammi, R.; Ochterski, J. W.; Martin, R. L.; Morokuma, K.; Farkas, O.; Foresman, J. B.; Fox, D. J. Gaussian 16 Revision C.01, 2016.

(7) Lu, T.; Chen, F. Multiwfn: A Multifunctional Wavefunction Analyzer. *J. Comput. Chem.* **2011**, *33* (5), 580–592. https://doi.org/10.1002/jcc.22885.

(8) Case, D.; Betz, R.; Botello-Smith, W.; Cerutti, D.; Cheatham III, T.; Darden, T.; Duke, R.; Giese, T.; Gohlke, H.; Goetz, A.; Others. AmberTools 16. *Univ. Calif. San Franc.* **2016**.

(9) Wang, J.; Wolf, R. M.; Caldwell, J. W.; Kollman, P. A.; Case, D. A. Development and Testing of a General Amber Force Field. *J. Comput. Chem.* **2004**, *25* (9), 1157–1174. https://doi.org/10.1002/jcc.20035.

(10) Jorgensen, W. L.; Chandrasekhar, J.; Madura, J. D.; Impey, R. W.; Klein, M. L. Comparison of Simple Potential Functions for Simulating Liquid Water. *J. Chem. Phys.* **1983**, *79* (2), 926–935. https://doi.org/10.1063/1.445869.

(11) Sousa da Silva, A. W.; Vranken, W. F. ACPYPE - AnteChamber PYthon Parser interfacE. *BMC Res. Notes* **2012**, *5* (1), 367. https://doi.org/10.1186/1756-0500-5-367.

(12) Abraham, M. J.; Murtola, T.; Schulz, R.; Páll, S.; Smith, J. C.; Hess, B.; Lindahl, E. GROMACS: High Performance Molecular Simulations through Multi-Level Parallelism from Laptops to Supercomputers. *SoftwareX* **2015**, *1–2*, 19–25. https://doi.org/10.1016/j.softx.2015.06.001.
